# A DNA Sequence Directed Mutual Transcription Regulation of HSF1 and NFIX Involves Novel Heat Sensitive Protein Interactions

**DOI:** 10.1371/journal.pone.0005050

**Published:** 2009-04-01

**Authors:** Umashankar Singh, Erik Bongcam-Rudloff, Bengt Westermark

**Affiliations:** 1 Department of Genetics and Pathology, Uppsala University, Uppsala, Sweden; 2 Linnaeus Center for Bioinformatics, Uppsala University, Uppsala, Sweden; Cinvestav, Mexico

## Abstract

**Background:**

Though the Nuclear factor 1 family member NFIX has been strongly implicated in PDGFB-induced glioblastoma, its molecular mechanisms of action remain unknown. HSF1, a heat shock-related transcription factor is also a powerful modifier of carcinogenesis by several factors, including PDGFB. How HSF1 transcription is controlled has remained largely elusive.

**Methodology/Principal Findings:**

By combining microarray expression profiling and a yeast-two-hybrid screen, we identified that NFIX and its interactions with CGGBP1 and HMGN1 regulate expression of HSF1. We found that CGGBP1 organizes a bifunctional transcriptional complex at small CGG repeats in the HSF1 promoter. Under chronic heat shock, NFIX uses CGGBP1 and HMGN1 to get recruited to this promoter and in turn affects their binding to DNA. Results show that the interactions of NFIX with CGGBP1 and HMGN1 in the soluble fraction are heat shock sensitive due to preferential localization of CGGBP1 to heterochromatin after heat shock. HSF1 in turn was found to bind to the NFIX promoter and repress its expression in a heat shock sensitive manner.

**Conclusions/Significance:**

NFIX and HSF1 exert a mutual transcriptional repressive effect on each other which requires CGG repeat in HSF1 promoter and HSF1 binding site in NFIX promoter. We unravel a unique mechanism of heat shock sensitive DNA sequence-directed reciprocal transcriptional regulation between NFIX and HSF1. Our findings provide new insights into mechanisms of transcription regulation under stress.

## Introduction

Nuclear factor 1 family of genes codes for site-specific DNA-binding proteins known to have multiple roles in replication, signal transduction and transcription [1]. Four known members of the family, NFIA, NFIB, NFIC and NFIX in higher eukaryotes, are evolutionarily highly conserved. No NFI gene is known in unicellular organisms indicating the importance of NFI genes in complex metazoan biology [2]. NFI proteins contain an N-terminal MH-1 DNA-binding domain and a C-terminal CTF-1 transcription modulation domain, which allows them to interact with other proteins [3], [4]. They bind as homodimers or heterodimers to TTGGC(N5)GCCAA sites and can also bind to either half of the palindrome as monomers [5]–[7]. The NFI proteins have highly similar peptide sequences and might have some redundant functions. Mouse knockout studies show that NFIA mutation results in hydrocephalus and abnormal brain development [8], NFIB mutation causes retarded lung development [9] and NFIC mutation causes abnormal tooth development [10], with very little overlap in phenotypes. NFIX knockouts generated by different groups have resulted in different phenotypes; hydrocephalus and abnormal ossification in one case [11] and defects in hippocampal development, neural stem cell differentiation, and weight-loss in the other [12]. Thus, other members of the family do not compensate for the functions of individual NFI genes. There are very few reports on the mechanisms of action of human NFI proteins. NFI protein overexpression results in resistance of chicken cells to transformation by *qin*, *jun* and *fos* oncogenes [13]. NFIC interacts with histone H1 [14], PIRIN [15] and TAFII55 [16] proteins and activates transcription at specific loci, such as glucocorticoid-responsive MMTV promoter [17], [18]. NFIX is important for activation of GFAP transcription in astrocytes [19] and provides resistance against TGFB-induced apoptosis in mink epithelial cells [20]. At the CDKN1A promoter, all different NFI members exhibit different levels of transcriptional repression [21]. This and different knockout phenotypes of NFI genes indicate the existence of mechanisms specific for each member. Differential interactions with other proteins, conferred by different post-transcriptional modifications and subtle differences in peptide sequences might result in functional specificities of different NFI members.

Retroviral tagging using MMULV expressing PDGFB to identify novel glioma-causing genes gave one integration in NFIA, NFIB and NFIC each and five integrations in NFIX [22]. Despite such strong indications of involvement of NFIX in PDGF-induced tumorigenesis, no systematic study has been undertaken to address molecular mechanism of action of NFIX.

Heat shock factor 1 (HSF1), a key regulator of heat shock-induced transcription [23], [24] is a potent modifier of carcinogenesis induced by a wide range of factors, including PDGFB [25]. Heat shock-induced misfolding of proteins leads to induction of chaperone activity and expression, which tends to rectify the errors in protein folding [26], [27]. Following heat shock HSF1 gets recruited to heat shock elements in the promoters of its target genes, and activates transcription [28], [29]. Unlike normal cells, tumor cells have higher proteotoxic stress and require higher levels of chaperones to survive [23]. While it is known that heat shock and protein denaturation induces HSF1 expression, the exact molecular mechanisms behind HSF1 transcriptional regulation are not known.

In this study, we describe NFIX peptides for the first time and report that NFIX regulates expression of stress related genes including HSF1. We identify that NFIX exists in a heat sensitive complex with CGGBP1 and HMGN1. CGGBP1 organizes a transcription regulatory complex comprising of NFIX and HMGN1 at a small CGG repeat element in the HSF1 promoter and suppresses its expression. HSF1 was also found to repress NFIX expression by binding to a potential HSF1-binding site in the NFIX promoter. We report a unique DNA sequence-directed reciprocal transcription regulatory mechanism between NFIX and HSF1 involving heat shock-sensitive protein interactions.

## Results

### NFIX regulates genes involved in stress response

To identify the functions of NFIX, we suppressed it by transient siRNA transfections. By real time quantitative RTPCR (qRTPCR) we confirmed that NFIA, NFIB and NFIC were not affected (not shown). As an NFIX peptide has never been demonstrated before, we also characterized the NFIX peptide and its intracellular localization. We also found that tyrosine phosphorylation affects intracellular localization of NFIX (Figure S1, Figure S2, Figure S3 and supplementary results in file Results S1). Thus we identified a 60 KDa nuclear peptide which could be immunoprecipitated and was down-regulated by NFIX-siRNA.

To detect transcriptional targets of NFIX, total RNA from control- or NFIX-siRNA transfected U-251 MG cells were profiled using cDNA microarrays in six pairs of dye-swap hybridizations representing six different transfection experiments (Figure S4). Using a very stringent B value threshold (a Bayesian approach to calculate log-odds of a gene being truly differentially expressed, [30], [31], we found 150 different ESTs being differentially expressed with a very high level of significance (Table S1). Of these, 55 were down-regulated and 95 up-regulated by NFIX-siRNA (Table S1) suggesting that NFIX has more repressive effect on transcription than activating.

While the genes with altered transcript levels were involved in diverse cellular processes (Table S1), the largest functional group of the these genes coded for proteins involved in unfolded protein response, such as HSPA1A, HSPA8 HSP90B1, XBP1, ATF4, PDIA4, DDIT3, SYVN1, TXNDC4, DERL2, HYOU1 and HERPUD1. The changed expression of these genes was confirmed by qRTPCR in the same RNA samples used for microarray hybridizations (not shown). To check if the regulation of stress-related genes by NFIX is a more general phenomenon or just limited to a specific cell type, we extended the qRTPCR-based expression analysis for a set of 11 genes (consisting of some stress-related genes and some involved in different functions) in U-251 MG (new transfections), U-2987 MG, U-343 MG-Cl2:6, U-1242 MG, U-87 MG and U-2197 cells (Table 1). NFIX-siRNA strongly down-regulated NFIX expression at the RNA level in all the cell types. Changes in the expression of the 11 candidate genes widely varied between different cell types with the direction of change of expression being randomly different from what we observed in microarray hybridizations (Table 1). HSF1 is a key regulator of heat shock chaperone expression and we explored if the NFIX-mediated regulation of stress response genes involves HSF1. NFIX-siRNA increased HSF1 transcript levels in all the cell lines except U-343 MG-Cl2:6 and U-251 MG (new transfections). In the RNA samples from U251 cells used for microarray hybridizations however, HSF1 expression was induced more than 1.8 folds after NFIX siRNA treatment (nor shown). Since the different batches of U251 cells exhibited different response towards NFIX siRNA, we used U-2987 MG cells for further experiments. The highly variable changes in expression patterns of NFIX target genes indicated that in different cells NFIX regulates stress pathways differently. Nevertheless, in some parts the variability in gene expression patterns caused by NFIX-siRNA may be due to differences in events downstream to HSF1 induction as HSF1 was affected by NFIX-siRNA in all cell lines, except in U-343 MG-Cl2:6 and somewhat inconsistently in U-251 MG. Thus, irrespective of the differences in gene expression patterns, it was confirmed that HSF1 is a target for NFIX in many cell lines.

**Table 1:** qRTPCRs in different cell lines on a panel of genes exhibiting altered transcript levels in the microarray expression screen in U-251 MG cells after NFIX-siRNA treatment.

	U-251 MG	U-343 MG	U-1242 MG	U-87 MG	U-2987 MG	U-2197
**NFIX**	**0.24±0.01**	**0.2±0.03**	**0.23±0.03**	**0.19±0.02**	**0.05±0.003**	**0.08±0.01**
DDIT3	2.15±0.24	-	1.32±0.15	1.44±0.21	4.38±0.81	2.54±0.038
DERL2	2.01±0.31	-	-	0.7±0.06	-	-
HERPUD1	3.09±0.47	-	1.38±0.2	-	-	-
HSPA1A	0.51±0.07	-	0.62±0.07	-	2.22±0.41	1.61±0.23
HSPA8	0.75±0.11	-	-	-	2.12±0.35	1.88±0.26
XBP1	3.15±0.52	-	-	1.28±0.11	1.93±0.44	2.39±0.35
PTRF	2.27±0.37	-	2.11±0.18	1.82±0.17	1.73±0.18	1.95±0.28
ADAM12	3.93±0.58	-	-	-	-	-
HYOU1	2.38±0.46	-	-	3.59±0.65	-	1.82±0.21
MMP2	2.79±0.39	-	-	1.62±0.27	-	-
**HSF1**	**1.2±0.14**	**1.02±0.04**	**1.73±0.22**	**1.96±0.29**	**1.87±0.27**	**2.21±0.42**
n =	6	6	4	4	8	4

Except for HSF1, only statistically significant (T-test, p value<0.05) changes in expressions are shown. Non-significant changes are shown as dashes. For HSF1, all changes are significant except U-251 MG and U-343 MG-Cl2:6. All values are average ±S.D. of the ratios of values for NFIX-siRNA over control-siRNA, calculated by delta-delta Ct method using GAPDH as internal control. For some reactions, ATCB was also used as internal control and similar values were obtained (not shown).

### NFI-binding sites do not dictate transcriptional regulation by NFIX

**Figure 1:**
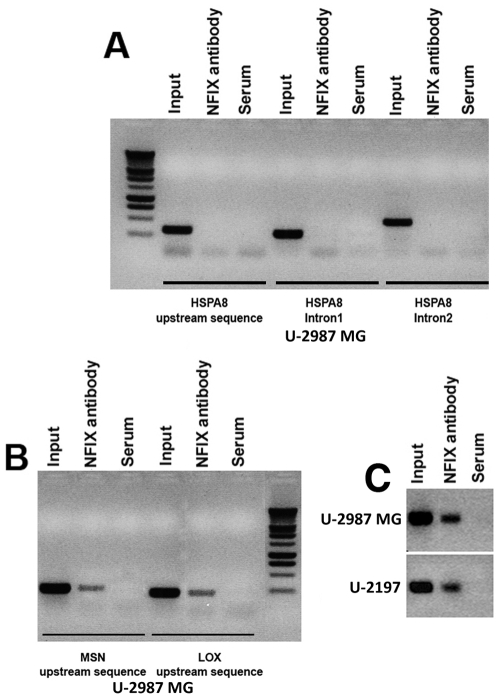
NFIX-DNA interactions at specific loci containing NFI-binding sites. NFIX does not bind to full NFI-binding sites in HSPA8 promoter or in the introns of HSPA8, which contain potential half NFI-binding sites (A). NFIX does bind to identical full NFI-binding sites in MSN and LOX promoters (primers flank the predicted NFI-binding site; refer to Table S2) (B). NFIX is recruited to the HSF1 promoter region devoid of any full NFI-binding sites (C).

Next we investigated if the transcriptional deregulation observed after NFIX-siRNA treatment is a direct effect of loss of NFIX binding to its target sequences or not. A TransFac search for commonly occurring transcription factor binding sites did not reveal any significant differences between the genes with altered transcript levels and a set of genes on the microarray which did not exhibit altered transcript levels (not shown). Since the NFI-binding site matrix used by TransFac is based on the half NFI-binding sites, we asked if the full palindromic NFI-binding sites existed in the promoter regions of genes with altered transcript levels. 3 Kb regions spanning upstream of the transcription start sites of 91 genes with altered transcript levels and 137 control genes (genes on microarrays, with least possibility of being differentially expressed) were extracted and searched for the presence of full NFI-binding sites, with different stringencies. The sequence motifs YGGM(N5)GCCAA, TGGM(N6)CCAA and TGGM(N6)CCA were found in 6.6, 17.6 and 53.8% of genes with altered transcript levels and in 2.2, 10.2 and 42.3% of control genes respectively (Table S2). Even if the genes with altered transcript levels consistently had a higher percentage of genes containing NFI-binding site in their promoters than the control genes, there was still a substantial number of genes on the microarrays, which were associated with NFI-binding sites and did not exhibit altered transcript levels. The genes with altered transcript levels HSPA8, MSN and LOX had NFI-binding sites in their promoters and NFIX binding to those sites in U-2987 MG cells was studied by chromatin immunoprecipitation (ChIP) PCR using NFIX antibody. NFIX was found to bind to the MSN and LOX promoters, but not the HSPA8 promoter (Figure 1). The HSF1 promoter was also analysed and was found to not harbour any full NFI-binding site but like many other promoters, some half sites were observed. Semiquantitative ChIP assays showed that NFIX bound to the HSF1 promoter in U-2987 MG and U-2197 cells (Figure 1). This showed that the presence of full NFI-binding site in promoter region was not a good predictor of altered transcription of a gene upon NFIX suppression by siRNA and that in a specific cell type, NFIX does not constitutively bind to all of its binding sites on DNA. Thus the transcriptional regulation by NFIX is mediated by additional mechanisms other than the known NFI-binding sites.

### NFIX interacts with CGGBP1 and HMGN1

Since there are no known interacting partners of NFIX which could be used as candidates to address this issue, we performed a yeast-2-hybrid screen of a fetal human brain cDNA library. Using the CTF1 domain of NFIX as bait, we screened approximately 10^7^ independent clones and identified two different prey clones corresponding to DNA-binding protein coding genes. These included a high mobility group protein (HMGN1) and the CGG triplet repeat binding protein 1 (CGGBP1) (Figure S5). HMGN1, a sequence-non-specific DNA-binding protein replaces histone H1 from nucleosomes and establishes open chromatin conformation associated with transcriptional activation [32] and the HSPA1A promoter is a proven target of HMGN1 [33]. CGGBP1 on the other hand is a transcriptional repressor binding to CGG triplet repeats [34]. *In vitro* binding assays have shown that 8 units of CGG repeats, even with a G-A mismatch, constitute a CGGBP1 binding site [34]. Naumann and coworkers found that CGGBP1 can bind to as small as five CGG repeats with one base mismatch [35]. CGGBP1 binding to DNA *in vivo* has never been studied at loci other than the FMR1 gene which has long CGG repeats in its 5′-UTR. The HSF1 promoter region is associated with a CpG rich region and we found that there is a 6 CGG tandem repeat (with just one G-A mismatch) spanning from −11 to +7 nucleotide bases relative to HSF1 transcription start site. This raised a possibility that CGGBP1 and HMGN1 might mediate transcriptional regulation of HSF1 by NFIX. The deregulation of many stress-response genes by NFIX suppression could thus be routed through HSF1.

### NFIX, CGGBP1 and HMGN1 regulate heat shock response genes in U-2987 MG cells

**Figure 2:**
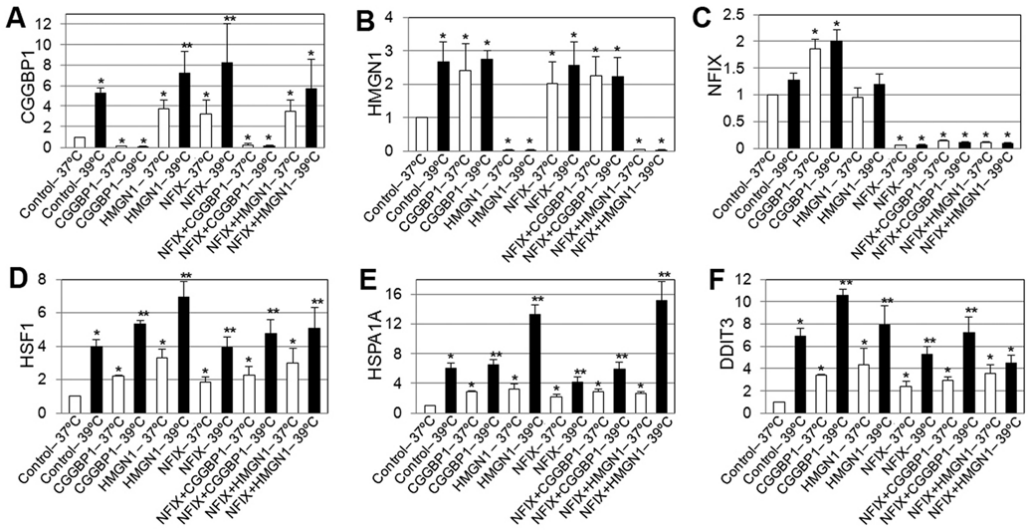
Transcription regulation of heat shock response genes by NFIX, CGGBP1 and HMGN1. CGGBP1 is induced by heat shock alone and depletion of HMGN1 or NFIX at 37°C. This induction by HMGN1 or NFIX depletion is further enhanced at 39°C (A). HMGN1 is induced by heat shock and depletion of CGGBP1 or NFIX at 37°C (B). NFIX is not induced by heat shock or HMGN1 depletion, but only by CGGBP1 depletion at 37°C and 39°C both (C). Bonafide heat shock response genes HSF1 (D), HSPA1A (E) and stress response gene DDIT3 (F) are induced by heat shock and all the siRNA combinations (D, E and F). Single asterisks indicate significant change compared to respective Control-37°C samples. Double asterisks indicate additional significance of change (p<0.05) due to heat shock compared to the respective samples at 37°C. The gene assayed is shown on the Y-axis and the different siRNA-temperature combinations are indicated along the X-axis. All values are average ±S.D. of ratios calculated by delta-delta Ct method using GAPDH as internal control with control-siRNA-37°C values as denominators. As for control-siRNA-37°C the value is this normalized to 1 in each case, no S.D. is shown.

If the interactions of NFIX with CGGBP1 and HMGN1 are important for its function, then the inhibition of these genes will recapitulate some of the effects observed after NFIX inhibition. With this premise, we tested if NFIX, CGGBP1 and HMGN1 also regulated transcription of NFIX target genes. U-2987 MG cells were cultured at 37°C or 39°C for 48 hours after five different kinds of siRNA transfections: control, CGGBP1, HMGN1, NFIX, CGGBP1 combined with NFIX, and, HMGN1 combined with NFIX. qRTPCRs were performed on RNA samples to check the expression of HSF1 and HSPA1A (heat shock response genes), DDIT3 (a stress response gene unrelated to heat shock response), CGGBP1, HMGN1 and NFIX, using GAPDH as control. siRNA against CGGBP1, HMGN1 and NFIX effectively silenced their respective target genes to extremely low levels similarly in samples incubated at 37°C or 39°C (Figure 2A, 2B and 2C). CGGBP1 expression was induced by HMGN1- and NFIX-siRNA, both separately and in combination at 37°C (Figure 2A). At 39°C, CGGBP1 was induced in the presence of control-siRNA whereas the inductions by HMGN1- or NFIX-siRNAs were further strengthened, compared to those seen at 37°C (Figure 2A). HMGN1 expression was induced similarly at 37°C and 39°C by siRNA against CGGBP1, NFIX or both combined. Also at 39°C, induction of HMGN1 expression was observed even by control-siRNA (Figure 2B). NFIX expression was induced only by CGGBP1-siRNA with no difference between the 37°C and 39°C samples (Figure 2C). HSF1 and its target gene HSPA1A were strongly induced by all the five siRNA combinations at 37°C and the induction was stronger at 39°C (Figure 2D, 2E). For DDIT3, induction was observed with all the five different siRNA combinations at 37°C, but the additional induction at 39°C was not observed in the combined NFIX- and HMGN1-siRNA sample (Figure 2F). These results showed that CGGBP1 and HMGN1 also regulate stress-related genes including HSF1 and thus the interactions of CGGBP1and HMGN1 with NFIX have functional significance in this context.

All of these genes except NFIX, CGGBP1 and HMGN1 are known stress-related genes and these results show that CGGBP1 and HMGN1 are both transcriptional regulators of heat shock response genes as well as transcriptional targets of heat shock themselves. Even if HSF1 induction was still a common feature of inhibitions of CGGBP1 and HMGN1, unlike CGGBP1-siRNA, HMGN1-siRNA did not induce NFIX expression. Since combining NFIX-siRNA with CGGBP1 or HMGN1-siRNA did not add up the effects of NFIX-siRNA with those of CGGBP1 or HMGN1-siRNA alone, a possibility was raised that transcriptional regulatory activities of CGGBP1 and HMGN1 involve NFIX and removal of either component caused same non-additive effect (Figure 2).

Despite some similarities in gene expression regulation by NFIX-, CGGBP1- and HMGN1-siRNA, we found some discrete phenotypes in U-2987 MG cells upon siRNA-mediated CGGBP1 and HMGN1 suppressions; such phenotypes were not observed after NFIX-siRNA treatment. Chronic heat shocking in presence of control-siRNA for 5 days resulted in elongation of cells (Figure S6), but a phenotypically identical effect was seen only after 2 days of CGGBP1-siRNA transfections at both 37°C and 39°C, with the effect being more pronounced at 39°C (Figure S6). Thus in the absence of CGGBP1 the cells responded both phenotypically and in terms of gene expression in a manner as if they were subjected to heat shock. HMGN1-siRNA on the other hand resulted in enlarged morbid cells at both normal and heat shock conditions (Figure S6). Just like the effect on HSF1 expression, combining siRNAs of NFIX with CGGBP1 or HMGN1 did not show any additive effect on these phenotypes (Figure S6). Overall, these results suggest that NFIX suppresses HSF1 transcription in a heat sensitive manner through pathways, which involve CGGBP1 and HMGN1.

### The soluble complex of NFIX, CGGBP1 and HMGN1 is heat shock sensitive

**Figure 3:**
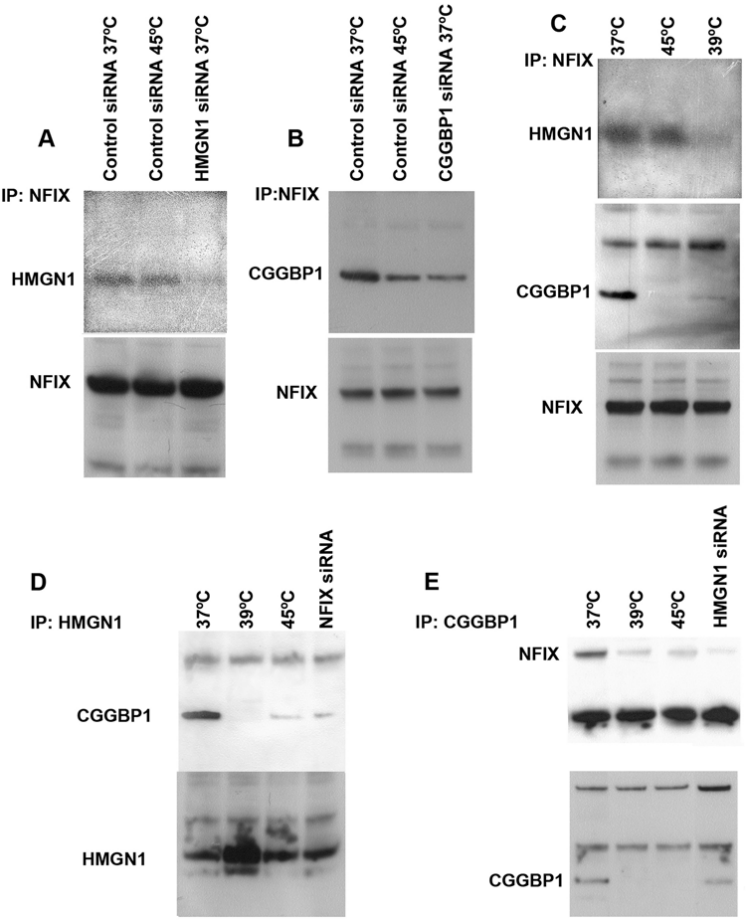
Interactions between endogenously expressed NFIX, CGGBP1 and HMGN1 in U-2987 MG cells. HMGN1 is immunoprecipitated with NFIX at 37°C, after 45°C-5 min heat shock and is specifically eliminated by HMGN1-siRNA (A). CGGBP1 is immunoprecipitated with NFIX at 37°C and is reduced by 45°C-10 min heat shock or CGGBP1-siRNA (B). HMGN1-NFIX interactions survive 45°C-10 min heat shock but not 39°C-48 h heat shock, whereas CGGBP1-NFIX interactions are sensitive to both acute and chronic heat shocks (C). HMGN1 and CGGBP1 interact at 37°C which is severely reduced by 39°C-48 h or 45°C-10 min heat shock and NFIX-siRNA (D). NFIX precipitated by CGGBP1 at 37°C is severely reduced by 39°C-48 h or 45°C-10 min heat shock and HMGN1-siRNA (E). NFIX levels were not affected by heat shock (A, B and C), HMGN1 levels were increased by 39°C-48 h heat shock (D), and CGGBP1 levels were decreased by heat shock (E).

The physical interactions of endogenously expressed NFIX with HMGN1 and CGGBP1 were confirmed by co-immunoprecipitation assays (Co-IPs) and the identities of HMGN1 and CGGBP1 bands were confirmed by using siRNA against them (Figure 3A, B). We then asked if interactions of NFIX with CGGBP1 and HMGN1 are constitutive or affected by heat shock. Co-IPs were performed by using NFIX antibody, on lysates of U-2987 MG cells, which were either cultured at 37°C, acute heat shocked at 45°C for 10 minutes or chronically heat shocked at 39°C for 48 hours, and were probed on Western blots using antibodies against CGGBP1 or HMGN1. CGGBP1 precipitated with NFIX was greatly reduced after acute heat shock and almost diminished after chronic heat shock (Figure 3B, C). There was no detectable difference in HMGN1-NFIX interactions following acute heat shock. However, chronic heat shock diminished this interaction too (Figure 3A, C). We then asked if NFIX interacts with CGGBP1 and HMGN1 only separately or also together in one complex. CGGBP1 was immunoprecipitated by the HMGN1 antibody and this was reduced by NFIX-siRNA and chronic heat shock (Figure 3D). Similarly, NFIX was immunoprecipitated by the CGGBP1 antibody and was sensitive to HMGN1-siRNA and chronic heat shock (Figure 3E). Thus it was proven that at least a significant fraction of endogenously expressed NFIX, CGGBP1 and HMGN1 exist in one heat shock-sensitive complex. The levels of precipitated NFIX and HMGN1 were unaffected by heat shock, when probed with same antibodies as used in co-IPs, however the levels of precipitated CGGBP1 were reduced after heat shock (Figure 3). Western analysis for CGGBP1 expression in the insoluble fraction of whole cell lysates from heat shocked U-2987 MG showed that CGGBP1 is not degraded upon heat shocking (not shown). This indicated that a fraction of these protein complexes get preferentially recruited to the insoluble fraction under heat shock and thus are lost in the cellular debris (which contains insoluble protein complexes and the genomic DNA with proteins tightly bound to it) thereby evading detection. We then addressed the effects of heat shock on sub-cellular localization of CGGBP1 and NFIX.

### NFIX and CGGBP1 localize to nuclei and stress granules

**Figure 4:**
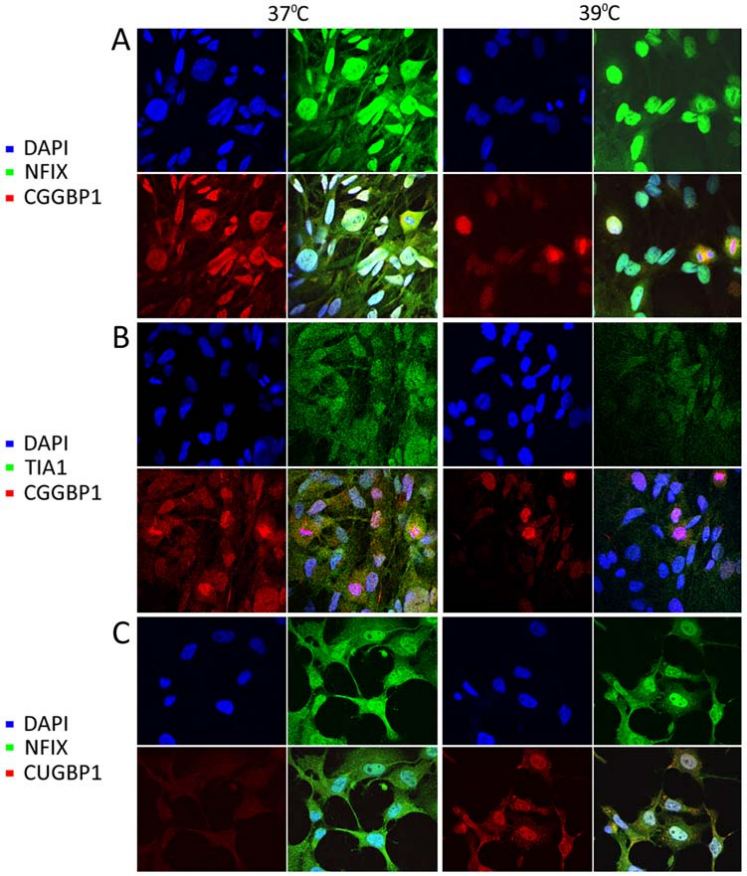
Immunofluorescence localization of NFIX and CGGBP1 in U-2987 MG cells after heat shock. Extranuclear presence of CGGBP1 and NFIX is reduced after heat shock (A). CGGBP1 co-localizes with TIA1 at 37°C but no preferential presence of CGGBP1 in stress granules is seen after heat shock (B). NFIX co-localizes with CUGBP1 after heat shock in both nuclei and cytoplasmic stress granules (C).

U-2987 MG cells either cultured normally or heat shocked at 39°C for 48 h were stained with NFIX and CGGBP1 antibodies. Both proteins were predominantly co-expressed in the nuclei both, after or without heat shock. However, in a subset of cells the extra-nuclear expression of both NFIX and CGGBP1 was strongly reduced and nuclear expression increased after heat shock (Figure 4A). Very strong presence of CGGBP1 was seen in strongly DAPI positive heterochromatin and this was further enhanced after heat shock (Figure S6). We also explored if CGGBP1 could also be present in stress granules, which are aggregates of prion-like insoluble complexes [36]–[38]. Using TIA1 and CUGBP1 as markers of stress granules, we found that TIA1 is present in very fine granules all over the U-2987 MG cells and CGGBP1 expression overlaps with it at 37°C (Figure 4B). However, after heat shock, TIA1-positive staining was reduced and CGGBP1 itself was seen prominently in the nucleus, suggesting that CGGBP1 might not be preferentially lost as a stress granule component after heat shock. After heat shock NFIX was found to co-localize with CUGBP1, another stress granule component, in the nucleus as well as in prominent granular structures in the cytoplasm (Figure 4C). These results suggest that though CGGBP1 and NFIX co-localize with components of stress granules, the loss of detection of CGGBP1 after heat shock in the soluble fraction of cell lysates is likely due to the its stronger heterochromatin-associated presence.

### NFIX depends on CGGBP1 and HMGN1 for binding to the HSF1 promoter

Next we investigated if and how CGGBP1, HMGN1 and NFIX interacted with the CGG repeat element containing the HSF1 transcription start site. U-2987 MG cells were transfected with control, CGGBP1-, HMGN1- or NFIX-siRNA and DNA-protein interactions at 37°C or 39°C were studied by ChIP-qPCR.

**Figure 5:**
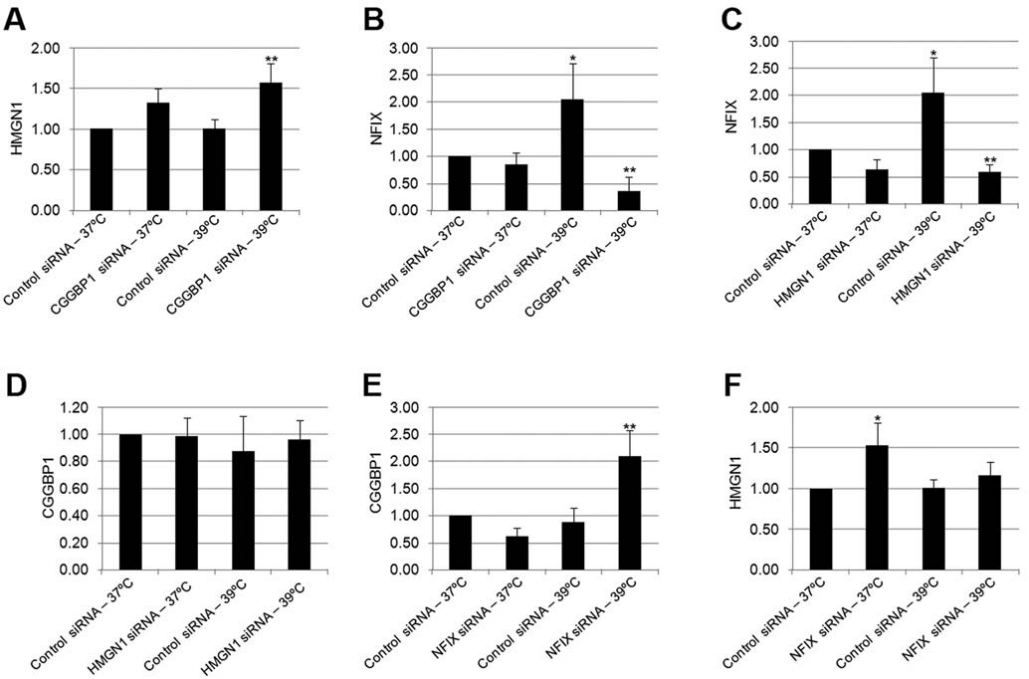
Dynamics of NFIX, CGGBP1 and HMGN1 occupancies at the HSF1 promoter in U-2987 MG cells. HMGN1 occupancy is increased by CGGBP1-siRNA at 39°C (A). NFIX occupancy is increased by heat shock alone and reduced by CGGBP1-siRNA at 39°C (B). Effect of HMGN1-siRNA on NFIX occupancy mimics the effects of CGGBP1-siRNA (C). HMGN1-siRNA or heat shock does not affect CGGBP1 occupancy (D). Loss of NFIX at 39°C increases CGGBP1 occupancy (E). Loss of NFIX at 37°C increases HMGN1 occupancy (F). Single asterisks indicate significant change (p<0.05) compared to respective control-37°C samples. Double asterisks indicate additional significance of change due to heat shock compared to the respective samples at 37°C. The protein assayed in ChIP is shown besides Y-axis and the different siRNA-temperature combinations are indicated along the X-axis. All values are average ±S.D. of ratios calculated by delta-delta Ct method using the input DNA for each sample as internal control with control-siRNA-37°C values as denominators. For control-siRNA-37°C the value is this normalized to 1 in each case, so no S.D. is shown.

CGGBP1-siRNA increased HMGN1 binding on the HSF1 promoter at 39°C and not at 37°C, while in presence of control-siRNA, heat shocking did not affect HMGN1 binding (Figure 5A). HMGN1, generally known as a transcriptional activator, is a suppressor of HSF1 expression in U-2987 MG cells (Figure 2D). So at 39°C, counteraction of HMGN1 binding to the HSF1 promoter by CGGBP1 may favour HSF1 transcriptional induction. This also showed that HMGN1 also binds to this region without CGGBP1. NFIX binding to the HSF1 promoter was severely reduced by CGGBP1-siRNA at 39°C, but not at 37°C (Figure 5B). Since in the presence of control-siRNA NFIX binding was increased at 39°C, this showed that heat shock increases NFIX recruitment to the HSF1 promoter in a CGGBP1-dependent manner. Thus CGGBP1 facilitates binding of NFIX, another HSF1 suppressor on the HSF1 promoter at 39°C. Hence, CGGBP1 organizes a bifunctional transcription regulatory complex at the HSF1 promoter in which it prevents and facilitates respectively two transcriptional repressors of HSF1; HMGN1 and NFIX. As interactions between CGGBP1 and HMGN1 or NFIX detectable in soluble fraction are largely lost at 39°C, this could be due to the ability of HMGN1 to bind more avidly to DNA when not complexed with CGGBP1 and on the other hand a strong dependence of NFIX on CGGBP1 to bind to the CGG repeats in the HSF1 promoter such that the heat shock surviving fraction of the CGGBP1-NFIX complex is tightly associated with specific DNA loci like the HSF1 promoter. Heat shock induced NFIX binding and this effect was lost by HMGN1-siRNA (Figure 5C) suggesting that NFIX binding to the HSF1 promoter is HMGN1 dependent in the same way as it is on CGGBP1. The effects of HMGN1-siRNA on NFIX binding recapitulated those of CGGBP1-siRNA. NFIX-CGGBP1 interaction is dependent on HMGN1, so the effects of HMGN1-siRNA could be due to the loss of interactions between NFIX and CGGBP1. HMGN1-siRNA alone or in combination with heat shock did not have any effect on CGGBP1 binding (Figure 5D). While heat shock did not affect CGGBP1 binding in the presence of control-siRNA, NFIX-siRNA strongly increased it at 39°C (Figure 5E), showing that at 37°C NFIX-CGGBP1 complex binds to the HSF1 promoter optimally, but at 39°C NFIX-free CGGBP1 can bind to the HSF1 promoter more efficiently than NFIX-CGGBP1 complex. HMGN1 binding was slightly increased at 37°C by NFIX-siRNA. At 39°C, neither control nor NFIX-siRNA had any effect on HMGN1 binding (Figure 5F). These results showed that (1) for binding to the HSF1 promoter CGGBP1 does not need NFIX or HMGN1 but NFIX does need CGGBP1 and HMGN1 strongly, (2) NFIX and CGGBP1 have mild inhibitory effect on HMGN1 binding to the HSF1 promoter at 37°C and 39°C respectively and (3) NFIX has a strong inhibitory effect on CGGBP1 binding to the HSF1 promoter at 39°C. Of all these three proteins, CGGBP1 is the only site specific DNA binding protein and the region we assayed in the HSF1 promoter contains a small CGG triplet repeat, suggesting that CGGBP1 directs this complex to the HSF1 promoter and under CGGBP1-siRNA treatment, this complex fails to be organized and recruited to the HSF1 promoter. CGGBP1 deficiency had opposite effects on HMGN1 and NFIX bindings at 39°C and NFIX deficiency had mild opposite effects on CGGBP1 and HMGN1 binding at 37°C. It seems that this transcriptional complex, stable and recruited to the HSF1 promoter under normal conditions, regulates the basal level of transcription and thus has bifunctional effects on HSF1 transcription.

### HSF1 binds to the NFIX promoter and regulates its expression in a heat shock sensitive way

**Figure 6:**
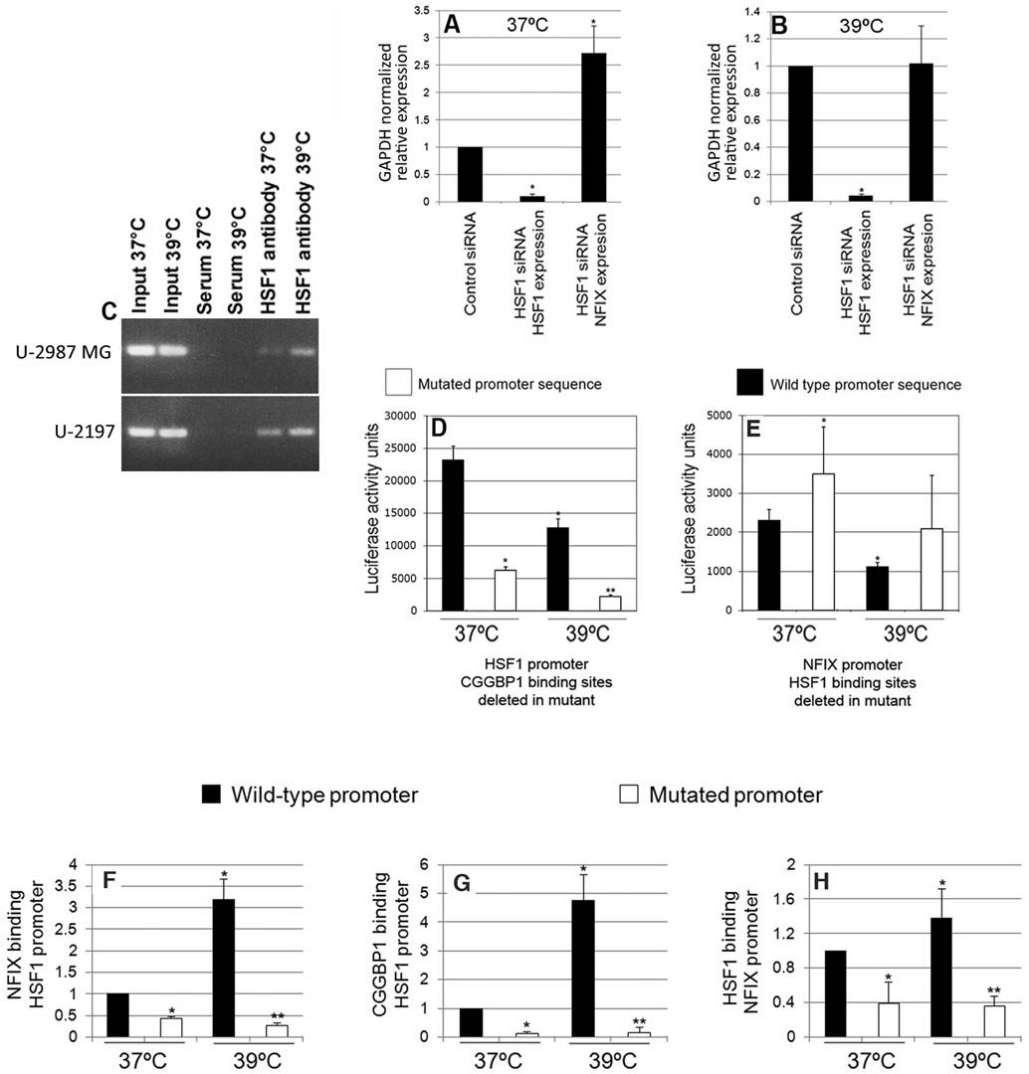
DNA sequence-dependent reciprocal transcriptional regulation between NFIX and HSF1. HSF1-siRNA increases NFIX expression in U-2987 MG cells at 37°C (A), which is lost at 39°C (B). HSF1 occupies the NFIX promoter in a heat shock dependent manner (C). High transcriptional activity of the HSF1 promoter is reduced strongly by both heat shock and loss of CGGBP1 binding sites (D). The NFIX promoter loses its transcriptional activity at 39°C significantly compared to 37°C and mutation of putative HSF1 binding sites causes increased transcriptional activity which is not significantly affected by heat shock (E). NFIX binding on the HSF1 promoter is increased by heat shock and this is lost by mutation of CGGBP1 binding site (F). Heat shock induced CGGBP1 binding is similarly affected by loss of its binding sites in the HSF1 promoter (G). HSF1 binding on the NFIX promoter is mildly induced by heat shock and mutation of HSF1 binding site reduces this binding (H). For all luciferase assays, HEK 293T cells have been used. Asterisks indicate significant change in expression (p<0.05) compared to control-siRNA-37°C in A and B, and, compared to wild-type promoter construct at 37°C in all other cases. Double asterisks indicate significant change compared to wild-type construct at 39°C. All values except D and E are average ±S.D. of ratios calculated by delta-delta Ct method using GAPDH or wild-type-37°C values as internal control. For the control-siRNA-37°C or wild-type construct-37°C, the value is this normalized to 1 in each case, so no S.D. is shown. In A and B, baseline values for HSF1 and NFIX using control-siRNA is shown by a single bar as the normalized value is 1 for both cases.

The mechanisms regulating NFIX transcription are largely unknown and from our results we know that CGGBP1 but not HMGN1 affects NFIX transcription (Figure 2C). There is however no CGG repeat in the NFIX promoter. Since we found that CGGBP1 regulates HSF1 expression, a possibility arose that the effect of CGGBP1-siRNA on NFIX transcription could be mediated by HSF1 and to test this we assayed NFIX transcription after RNAi against HSF1. Control or HSF1-siRNA was transfected in U-2987 MG cells and its effect on NFIX transcript levels was assayed by qRTPCR. Unlike control-siRNA, HSF1-siRNA increased NFIX transcript levels at 37°C (Figure 6A). At 39°C for 48 hours, this increase in NFIX transcript levels by HSF1-siRNA was lost (Figure 6B). HSF1 is a transcription factor and is known to bind to different combinations of inverted and non-inverted tandem (NGAAN) repeats with the spacer regions varying from 2 in non-inverted repeats to 7 in inverted repeats [39]–[43]. It is not known how slight variations of these consensuses affect HSF1 binding in human cells. The NFIX Ensembl transcript ENST00000264825 promoter has a potential minimal HSF1 binding site “GAAAAGAAAATGAA” from positions −585 to −572. We then examined if HSF1 indeed bound to this region in the NFIX promoter. Semiquantitative ChIP assays in this region using HSF1 antibody showed that HSF1 indeed bound to the NFIX promoter in U-2987 MG and U-2197 cells under normal culturing conditions and after chronic heat shock, HSF1 occupancy at the NFIX promoter increased (Figure 6C). This confirmed that (1) HSF1 is a heat shock-sensitive inhibitor of NFIX expression, and (2) HSF1 binds to the NFIX promoter in a heat shock dependent manner with a possible involvement of the putative HSF1 binding sites in the NFIX promoter. As HSF1 knockdown failed to induce NFIX expression after heat shock, it is implied that even in the absence of HSF1, other mechanisms control NFIX induction after heat shock.

### Specific binding sites for CGGBP1 in the HSF1 promoter and HSF1 in the NFIX promoter are required for normal transcriptional activities of the respective promoter elements

As suggested by our results, the mutual regulation of transcription of HSF1 and NFIX by each other involves targeting of transcription regulatory complexes at promoters of these two genes at the CGGBP1 binding site in the HSF1 promoter and a putative HSF1 binding site in the NFIX promoter. These sites were contained in the PCR-amplified region in ChIP experiments and so there was indirect evidence that these sites are indeed involved in CGGBP1 and HSF1 binding respectively. To establish if these sites were necessary for the binding of their respective proteins, we performed luciferase assays using the regions amplified in ChIP experiments as promoters, such that the CGGBP1 and HSF1 binding sites were either wild-type or deleted. In the NFIX promoter (a 234 bps region −404 bps to −171 bps relative to transcription start site), HSF1 binding site was mutated to an *NdeI* site. In the HSF1 promoter, the CGGBP1 binding site could not be site-specifically mutated due to extremely high GC richness and repetitive nature of this region. Instead, we deleted a 258 bps GC rich repetitive region, which included the CGGBP1 binding site. Thus, the wild type HSF1 promoter was a 466 bps region from −298 to +168 bps relative to the transcription start site whereas the mutant promoter was a 208 bps region from −298 to −91 bps relative to the transcription start site of the HSF1 gene. The basal activity of the NFIX wild type promoter was low but that of the HSF1 promoter was high. The mutations removing CGG repeat in the HSF1 promoter drastically decreased the transcriptional activity as the luciferase activity was reduced (Figure 6D) proving that this sequence element is important for the transcriptional activity of the HSF1 promoter. The mutation of the potential HSF1 binding site in the NFIX promoter increased luciferase activity (Figure 6E) and this was in line with the observation that HSF1 is a suppressor of NFIX expression. At 39°C the activities of the wild-type and mutant HSF1 promoters and the wild-type NFIX promoter were reduced, but the mutant NFIX promoter activity was not significantly affected (Figure 6E). ChIP assays with primers specific for luciferase construct regions flanking the cloned promoter revealed that these mutations severely reduced CGGBP1 and NFIX binding (Figure 6F, 6G) and mildly but significantly reduced HSF1 binding (Figure 6H) on their respective constructs, thereby proving that these specific candidate regions are indeed involved in transcriptional regulation and are required for CGGBP1 and HSF1 binding respectively at NSF1 and NFIX promoters. As HSF1 occupancy at the NFIX promoter was not absolutely dependent on the candidate region we studied, this might also involve other mechanisms.

## Discussion

In this study we have discovered that NFIX gene codes for two different peptides of which the larger peptide is localized in the nucleus and tyrosine phosphorylation affects its intracellular localization. Since tyrosine phosphorylation is one of the first steps in growth factor receptor signaling activated in tumorigenesis [44], our findings raise possibilities that NFIX could be a substrate for some oncogenic growth factor such as PDGFB, with which it has been shown to cooperate in glioma formation. Heat shock and stress related genes have emerged to be transcriptional targets of NFIX, of which HSF1 is very interesting. HSF1 emerged as a target of NFIX inhibition in all cell lines except U-343 MG-Cl2:6 and some variability in U-251 MG. The inertia of U-343 MG-Cl2:6 cells against any change in heat shock gene expression upon NFIX siRNA treatment could be either due to functional redundancy in the NFI family. Alternatively, it could be so that these cells are used to very low expression of NFIX (Figure S1C) and so lack of NFIX does not produce a significant effect on these cells. HSF1 deficiency has been shown to mitigate PDGFB-induced transformation [25]. Our results raise the possibility that growth factors could regulate HSF1 expression and tumorigenesis through tyrosine phosphorylation and nuclear localization of NFIX. Our experiments with NFIX-siRNA and chronic stress show that NFIX along with HMGN1 and CGGBP1 is an important regulator of HSF1 expression and stress response. Heat sensitive interactions of NFIX with CGGBP1 and HMGN1 and existence of all these three proteins in one transcription regulatory complex is a novel finding. Existence of CGGBP1 binding sites in the HSF1 promoter and recruitment of NFIX, CGGBP1 and HMGN1 to this site such that under chronic heat shock conditions CGGBP1 and HMGN1 become limiting for NFIX binding, shows that the integrity and stress-induced recruitment of the NFIX-CGGBP1-HMGN1 complex to the HSF1 promoter is tightly regulated. The CGGBP1 and HMGN1 dependence of NFIX binding to the HSF1 promoter may partly be due to heat sensitivity of interactions between NFIX, CGGBP1 and HMGN1. Similar heat-sensitive interaction between YWHAQ and FUSIP1 has been previously reported [45]. In addition, our results support the possibility that after heat shock a significant amount of CGGBP1 is strongly recruited to heterochromatic DNA thereby evading detection in the soluble fraction. The fact that NFIX and CGGBP1 co-localize with stress granule components sheds more light on the different mechanisms by which these proteins are involved in stress response. Our results also show that while NFIX is largely, if not absolutely, dependent on CGGBP1 for binding to the HSF1 promoter, HMGN1 does bind to this region even without CGGBP1. Moreover, opposite effects of CGGBP1-siRNA on NFIX and HMGN1 recruitment to the HSF1 promoter shows that these three proteins together constitute a bifunctional regulatory network of HSF1 transcription. Suppression of NFIX by HSF1 is a novel finding as is our observation that NFIX is a target of HSF1 and that HSF1 not only activates, but also represses transcription. Similarly, we report for the first time that HMGN1 can act as transcriptional repressor.

To test the function of CGG repeats in the HSF1 promoter, we deleted a 258 bps region from the wild-type promoter, as described in the results section. This deleted region contains 43 CpG dinucleotides which could potentially be methylation-dependent transcriptional regulatory elements for the HSF1 gene. CpG methylation is typically associated with silencing of gene expression [46] and loss of such elements could add to the transcriptional activity of HSF1 promoter region addressed by us. However, CpG methylation status of the the HSF1 promoter has not been reported to date. Loss of such a CpG-rich region could affect transcription not only through the removal of DNA methylation, but also by affecting the binding of other regulatory proteins in that region [46]. Since (1) this region contains bonafide CGGBP1 binding site(s), (2) CGGBP1 binds to this region, (3) CGGBP1 RNAi activates transcriptiuon from this promoter and (4) loss of this CpG-rich region abrogates CGGBP1 and associated NFIX binding to the DNA in the HSF1 promoter, CGGBP1 certainly is a major regulator of HSF1 expression through binding to this region in its promoter.

HSF1 expression is augmented by stress and accumulation of denatured proteins, but it is not known what mechanisms keep HSF1 transcription under check during normal and induced conditions. Our results show that NFIX, HMGN1 and CGGBP1 are repressors of HSF1 expression out of which HMGN1 and CGGBP1 are induced by heat shock whereas HSF1-regulation of NFIX is heat shock sensitive. It is known that sustained HSF1 expression leads to induction of the unfolded protein response, which culminates in apoptosis. Due to the inherently high proteotoxic stress in the tumor derived cells, inhibition of HSF1 induction might be a way to counteract the induction of unfolded protein response and avoid apoptosis; a feature that might be necessary for the cell survival. NFIX also emerged as a regulator of CGGBP1 and HMGN1 recruitment to the HSF1 promoter. Interestingly then, HSF1 expression in glioma cell line U-343 MG-Cl2:6 with low NFIX expression (Figure S1C) was not sensitive to NFIX-siRNA.

The regulation of NFIX transcription is enigmatic and has never been addressed before. Our attempts to establish stable NFIX over-expression systems in human glioma cell lines were unsuccessful and in U-251 MG cells, we did observe loss of cell division in cells transfected with NFIX expressing plasmid (Singh and Westermark, unpublished results). Even in inducible expression system for NFIX, established in U-251 MG and U1242 cell lines, we found extremely low levels of induction in several different clones (Singh and Westermark, unpublished results). This suggests that these cells do not tolerate high levels of NFIX and thus its transcription is under a very tight control, even under heat shock conditions. We identify HSF1 as one of the controllers of endogenous NFIX transcription. In the transgenic systems, where non-mammalian promoters drive NFIX expression, it might also be under post-transcriptional control. Interestingly, human NFIX contains a huge 3 prime UTR which is a target of different microRNAs. The mouse NFIX however lacks this long UTR element and it will be interesting to see the stress response in NFIX knock-out mice.

We thus report for the first time that heat shock-sensitive interactions between NFIX, CGGBP1 and HMGN1 mediate a DNA sequence directed inhibition of HSF1 transcription and in a unique mechanism of reciprocal transcription regulation, HSF1 also inhibits NFIX expression by using specific DNA sequence motifs.

## Materials and Methods

### Cell culture and siRNA transfections

Cells were cultured at 37°C, 5% CO2 in 10% FCS, 1% Glutamine and antibiotics supplemented minimum essential Eagel's medium (SIGMA). siRNA transfections were performed as per manufacturer's instructions using Dharmafect 2 and siRNA from Dharmacon. Sequences for siRNA are available on request. Before heat shocking (see Results S1), cells were left in transfection condition for 48 hours followed by medium change.

### ChIP assays

ChIP assays were performed with slight modifications to the ChIP protocol accompanying Upstate (Millipore) EZ ChIP reagents. Cells were cultured as required for each assay and fixed with 1% formaldehyde for 10 minutes at 37°C. Formaldehyde containing medium was promptly removed by two ice cold PBS washes, cells were lysed in SDS lysis buffer containing protease inhibitors and sonicated to fragment size ranging between 400 and 150 bp. Input was separated, samples diluted in ChIP dilution buffer and cleared with protein A-sepharpose beads for 1 h. 2 µg specific antibody or 5 µl rabbit serum was added to the samples, incubated overnight at 4°C followed by 1 h with the beads. Beads were washed with increasing salt concentration buffers, chromatin eluted by SDS-bicarbonate buffer and samples were decrosslinked at 65°C in presence of high salt concentration. DNA was purified by phenol-chloroform method, precipitated and used for PCR assays. All samples were processed identically and equal volumes of samples were taken for PCR assays. Since different quantities of DNA can be precipitated by same antibody under different treatments, DNA was not quantitatively equalized for each sample. Input was used as control for amount of chromatin subjected to ChIP.

### Real time PCRs and qRTPCRs

Total RNA was isolated and cDNA was synthesized using reverse transcription kit (New England Biolabs). ChIP DNA (using specific antibody, rabbit serum as negative comtrol) and input DNA were obtained as described above. Equal amounts of cDNA and equal volumes of ChIP DNA were used as template for each assay. GAPDH was used as internal control in all qRTPCRs and ACTB was used an additional internal control in some cases. All reactions were performed using SYBR-green premix (Applied Biosystems) and values calculated using delta-delta Ct method. Melt curve analysis (50°C to 99°C were performed to establish specificity of amplification). Values for serum negative controls are not shown in any graph because the Ct values were too high or not obtained. All primer sequences are mentioned in Table S3.

Other methods are described in Results S1.

## Supporting Information

Results S1 Characterization of molecular weights of NFIX peptides, an antibody against NFIX and tyrosine phosphorylation sites in NFIX.
(0.08 MB DOC)

Figure S1 Different NFIX peptides: (A) C-terminal FLAG tag recognizes an 18 KDa peptide coded by NFIX-C-FLAG, which together with 8 KDa tag is recognized as a 26 KDa band. (B) N-terminal HA tag recognizes a 60 KDa peptide coded by HA-N-NFIX. (C) Rabbit anti-human NFIX antibody directed against C terminus of NFIX peptide recognizes both the peptides in nine different human glioma cell lines. U-2987 MG expresses highest and U-343 MG expresses lowest amounts of the 60 KDa peptide. Comparable protein loading in different lanes was confirmed by Coomassie blue staining (not shown). (D) HA-N-NFIX codes for both peptides as its transfections increase expression of both peptides as recognized by NFIX antibody. (E) NFIX antibody recognizes the 60 KDa peptide but not the 26 KDa peptide coded by NFIX-C-FLAG construct, perhaps due to epitope masking by FLAG tag. (F) NFIX-siRNA downregulates both peptides in U-2987 MG cells, confirming the identity of the two peptides as products of NFIX mRNA. The last lane shows NFIX expression in HEK 293T cells, used in transient transfection experiments. Two lanes for each construct shown in (A), (B), (D) and (E) represent the expression from two independent clones for each construct.
(1.33 MB TIF)

Figure S2 Expression of NFIX peptides in U-251 MG cells. (A) C-terminal FLAG tagged peptide is primarily present out of nucleus (top two panels). Rarely, it was seen strongly in nucleus also (bottom panel). (B) N-terminal HA-tagged peptide is nuclear in most cells (top two panels) and infrequently present in cytoplasm too (bottom panel). (C) NFIX antibody recognized endogenously expressed NFIX as present all over the cells. Staining with no primary antibody and pre-immune serum in case of NFIX antibody were used as negative controls.
(2.38 MB TIF)

Figure S3 Tyrosine phosphorylation of NFIX. (A) Endogenously expressed NFIX is tyrosine phosphorylated in U-251 MG and U-2987 MG cells. (B) Transgenically expressed wild type (WT) and Y151F mutant constructs showed comparable levels of phosphorylation. But Y253F mutation was associated with reduction in phosphorylation levels with comparable levels of expression of NFIX peptide irrespective of the mutations. The HA antibody non-specifically recognized an approximately 90 KDa peptide. IP; immunoprecipitation, pY; phosphotyrosine antibody, Probe; the primary antibodu used in Western analysis following IP. (C) and (D); Y151F mutation does not affect intra-nuclear presence of HA-N-NFIX peptide (top panels of C and D); Y253F mutation leads to extra-nuclear presence of a fraction of HA-N-NFIX (lower panels of C and D). See text for details of the constructs.
(1.21 MB TIF)

Figure S4 Heat-map plot of 12 microarray hybridizations show reproducibility of NFIX-siRNA-induced changes in global gene expression. The hybridizations represented by transfection number 6 are the most different from the remaining 5 for most of the reporters.
(1.46 MB TIF)

Figure S5 Sequence alignment of prey clones identified in the yeast-2-hybrid screen with RefSeq RNA sequences. (A) Two different clones of HMGN1 and (B) one clone of CGGBP1. Stars indicate exact match.
(0.02 MB PDF)

Figure S6 Phenotypes of U-2987 MG cells after chronic heat shock and/or siRNA indicated. CGGBP1-siRNA recapitulates chronic heat shock even at 37Â°C and HMGN1-siRNA leads to enlarged morbid cells. Combining either siRNA with NFIX-siRNA did not worsen the phenotypes.
(2.09 MB TIF)

Table S1 List of 150 deregulated reporters (spots representing genes on the microarrays; in genes are represented by more than one reporter) in all six pairs of hybridization with a very high statistical significance (p<0.01). The Genbank ID, GeneOntology functional categories and other relevant information are provided. High B values show higher probability of being differentially expressed.
(0.08 MB XLS)

Table S2 Results of search for different full NFI binding sites in the 3 Kb upstream sequences from transcription start sites of 97 deregulated genes and 137 control genes. The deregulated genes are those with very high B value (B value>1, p value<0.025) as shown in table S1. The control genes are the genes represented on the microarrays with lowest B values (from the bottom of the list of all genes on the microarray, arranged by decreasing probability of being differentially expressed). Only for these numbers of genes reliable sequence could be obtained and only matches are shown.
(0.05 MB XLS)

Table S3 Sequences of oligonucleotides used for PCR, cloning, site-directed mutagenesis and siRNA experiments.
(0.03 MB XLS)
