# Supplementary material for: A DNA Sequence Directed Mutual Transcription Regulation of HSF1 and NFIX Involves Novel Heat Sensitive Protein Interactions
Source: PLoS One. 2009 Apr 1;4(4):e5050. doi: 10.1371/journal.pone.0005050 (PMC2660424; doi:10.1371/journal.pone.0005050)
Supplement: Results S1 — Characterization of molecular weights of NFIX peptides, an antibody against NFIX and tyrosine phosphorylation sites in NFIX. (0.08 MB DOC) [file pone.0005050.s001.doc]

**Supplementary results**

Human NFIX gene encodes two different peptides

Human NFIX transcripts have been characterized and described; yet experimental analysis of human NFIX peptide has never been reported. Several different transcripts are known to originate from human NFIX locus and. The coding region of human NFIX cDNA (NCBI RefSeq ID NM_002501.2; Ensembl ID ENST00000360105), predicted to code for a 49 KDa peptide, was used in all of our experiments. When we started our investigation no antibody was commercially available against human NFIX, so we generated NFIX cDNA clones with different tags. We cloned NFIX with a C-terminal FLAG tag (NFIX-C-FLAG), transiently transfected it in HEK293T cells and in Western analysis with anti-FLAG antibody found a specific band at ≈ 26 KDa (Figure S1A). The FLAG tag in this construct weighs 8 KDa so the molecular weight of the NFIX peptide thus detected is 18 KDa, much smaller than the predicted 49KDa. We generated an N-terminal HA-tagged NFIX construct (HA-N-NFIX) and in this case the tag accounted for less than 1 KDa. Unlike the empty vector, the HA-N-NFIX transfections specifically gave a ≈ 60 KDa band (Figure S1B). We then made a rabbit anti-human NFIX antibody against amino acids 422-435 of peptide NP_002492.2. We selected this region by predicting antigenicity as described elsewhere (Hopp and Woods, 1981). Lysates from 9 different human glioma cell lines when probed with NFIX-antiserum detected two different bands at 18 KDa and 60 KDa (Figure S1C). In all the cell lines the 18 KDa peptide had higher expression than the 60 KDa peptide. Transfection of HA-N-NFIX construct led to an increase in expression of both peptides when probed with NFIX antibody (Figure S1D). Transfection of the NFIX-C-FLAG construct increased the expression of the 60 KDa peptide but did not show a 26 KDa peptide when probed with NFIX antibody (Figure S1E). This could be due to the masking of the small C-terminal epitope by the large FLAG tag. To further establish the fact that these two peptides indeed originated from the NFIX gene, we used a combination of three different siRNA directed against different regions of NFIX transcript. By real-time quantitative reverse transcription PCR (qRTPCR) we first established that this siRNA did not down-regulate NFIA, NFIB and NFIC. Using sequence non-specific siRNA as control, it was found that the NFIX siRNA down-regulated both the peptide forms (Figure S1F) proving that both, the endogenous NFIX gene, as well as the NFIX transgenes with different tags, give rise to two different peptides of molecular weights 18 KDa and 60 KDa. However, the N-terminal tag is retained only with the larger peptide and the C-terminal tag is retained with only the smaller form.

We then performed immunofluorescence in U251 and 18KJ40 cells transfected with HA-N-NFIX and NFIX-C-FLAG constructs using antibodies against HA tag, FLAG tag or endogenous NFIX. Intriguingly, the 60 KDa HA-tagged NFIX was detected strongly in the nucleus (Figure S2A) while the smaller 18 KDa FLAG-tagged form was detected strongly outside the nucleus (Figure S2B). In some transfected cells however, the expressions were seen all over the cells (Figure S2A, S2B). Antibody against endogenous NFIX detected very strong expression in both, nuclei and cytoplasm (Figure S2C). In all further experiments, only the 60 KDa peptide is addressed because the 18 KDa peptide was detected in the western blots only when uncleared whole cell lysates were loaded on the gel, including the debris. For immunoprecipitations performed in many experiments we needed to clear the lysates first, so we could never immunoprecipitate the 18 KDa peptide.

Tyrosine phosphorylation affects intracellular localization of NFIX

NFIX was discovered as a PDGFB cooperating gene in glioma genesis and tyrosine phosphorylation is a key step in PDGF signalling. An *in silico* analysis by a previously published method (Blom et al., 1999) revealed that the tyrosine residues at positions 151 and 253 in NFIX are very strong candidates for being phosphorylated. Immunoprecipitation using NFIX and anti-phosphotyrosine antibodies showed tyrosine phosphorylation of NFIX in U251 and 18KJ40 cells (Figure S3A). The tyrosine residues 151 and 253 in the HA-N-NFIX construct were mutated to phenylalanine and three different types of mutant constructs were generated: HA-N-NFIX-Y151F, HA-N-NFIX-Y253F and HA-N-NFIX-Y151,253F. Immunoprecipitation with HA antibody on HEK293T cells transfected with these constructs showed that only the Y253F mutation caused reduction of phosphorylation (Figure S3B). Combined Y151-253F mutations did not completely remove the tyrosine phosphorylation (Figure S3B), suggesting that other tyrosines in the NFIX peptide are also phosphorylated.

Immunofluorescence performed with HA antibody on U251 and 18KJ40 cells transfected with HA-N-NFIX and three tyrosine mutant constructs showed that the HA-N-NFIX Y151F peptide was present in the nucleus (Figure S3C). The Y253F mutations resulted in extra-nuclear presence of the NFIX peptide with only very low presence in the nucleus (Figure S3D) suggesting that phosphorylation of tyrosine 253 is a determinant of nuclear localization of NFIX. The rarely seen extranuclear 60 Kda NFIX peptide in U251 and 18KJ40 cells could be the fraction not phosphorylated at Y253. It implies that the most of the 60 KDa NFIX peptide is phosphorylated in U251 and 18KJ40 cells.

**References**

Hopp, T.P., and Woods, K.R. (1981). Prediction of protein antigenic determinants from amino acid sequences. Proc Natl Acad Sci U S A *78*, 3824-3828.

Blom, N., Gammeltoft, S., and Brunak, S. (1999). Sequence and structure-based prediction of eukaryotic protein phosphorylation sites. J Mol Biol *294*, 1351-1362.

**Supplementary materials and methods**

Microarray hybridizations and analysis

Total RNA was extracted using Trizol (Invitrogen), quality assessed by agarose gel electrophoresis and 260/280 nm > 1.9. Labeling and hybridizations were done on Human 19K arrays from University health Network, OCI, Canada using TSA-Micromax kit (NEN-Perkin Elmer) with slight modification to the prescribed protocol. Arrays were scanned using Scanarray Express V 2.1 (Packard Biosciences). Raw data were exported as GPR files and subjected to within array print-tip LOWESS normalization. Data from all hybridization were pooled in to calculate the log odds of each reported being differentially expressed using B statistics (Gottardo et al., 2003; Lonnstedt and Speed, 2002; see references for detailed citations). Reporters with B value > 1 on all 12 hybridizations were regarded as truly differentially expressed. All raw and processed data are stored and were analyzed using microarray analysis tools developed by Linnaeus Center for Bioinformatics, Uppsala University, Uppsala.

Immunoprecipitations

Cells were cultured and heat shocked as required for each experiment and lysed using phosphate buffered RIPA and cleared of debris and DNA by centrifugation and incubated with 2 mg antibody overnight at 4ºC followed by Protein A sepharose beads (GE Life sciences) for 1 h. Beads were washed using RIPA buffer 3 times, eluted and processed for Western blot using NuPAGE reagents and protocol (Invitrogen)

Luciferase assays

Promoter elements were PCR amplified and cloned in pGL4.12 (Promega) in *KpnI* and *XhoI* sites, and equal amounts of DNA were transfected in triplicates in identically seeded and cultured HEK293T cells for 48 h. Luciferase kit substrates A and B (BD Biosciences) were added to lysates of transfected cells and immediately luminescence was measured according to manufacturer’s instructions.

Yeast-two-hybrid screen

Full-length NFIX cDNA or NFIX cDNA without MH-1 DNA-binding domain was cloned in *NdeI* and *SalI* sites in pGBKT7 vector and transformed into AH109 yeast strain. The full length NFIX gave background MEL1 activation on X-alpha-gal plates, so the clone without the DNA-binding domain was used used as bait. All reagents were purchased from Clontech. 10^7^ clones of a human fetal brain cDNA libraray cloned in pACT2 were screened for interactions by co-transformation and screening on adenine, leucine, tryptophan, histidine deficient plates containing X-alpha-gal. 13 blue colonies were serially selected on maximum stringency plates for 12 weeks, plasmids extracted and transformed in bacteria and sequenced as PCR products.

Site-directed mutagenesis

The site-directed mutagenesis to mutate the two tyrosine sites in NFIX to phenylalanine and for mutating potential HSF1 binding site in NFIX promoter to *NdeI* site, QuickChange II kit (Stratagene) was used.

NFIX cDNA cloning and plasmid transfections

Fulllength NFIX cDNA was amplified from cDNA prepared from U251 cells and cloned into pcDNA3.1+ (Invitrogen) into *KpnI* and *XhoI* sites with an N-terminal 1xHA-tag. For C-terminal FLAGx3 tagging, the same fragment was cloned into *EcoRV*-*HindIII* sites of pIRES-hrGFPII vector (Stratagene). All plasmid transfections were performed using Fugene reagent (Roche).

NFIX antibody development

The C-terminal fragment of NFIX peptide was synthesized and conjugated with keyhole lymphocyte antigen. 200 mg of peptide was injected into rabbit and serum collected either before injection or after 20 days of injection. Booster doses were injected every 20 days and serum collected. The same peptide was used to purify the NFIX antibody from the serum using antibody purification kit (Epitomics). The pre-immune serum and NFIX siRNA was used as negative controls to characterize the specificity of the antibody.

Statistics

Two-tailed heterocedastic T-test was performed in Microsoft Excel on raw values and statistical significance was assumed for p < 0.05. All values are presented as average ± standard deviation.

Heat shock

All heat shocks were performed after at least 48 hours of seeding the cells (in experiments were transfection were not required) and after 48 h and medium change of siRNA transfections in experiments requiring transfections. Acute heat shock was performed in an ordinary incubator (no CO2 or humidity control) at 45ºC for 5, 10, 20 or 30 minutes. Chronic heat shock was performed in standard cell culture CO2 incubators at 39ºC for 48 h. Cells were immediately processed after heat shock for further steps of the experiments.

Western blotting

All Western blotting experiments were performed using NuPAGE reagents Invitrogen). All gels were run on 4-12% Bis-Tris gels using MES running buffer. The gel showing FLAG-tagged NFIX was run using MOPS running buffer. All blots were performed in denaturing conditions. For NFIX antibody, 20% fetal calf serum was used as blocking buffer. For all other antibodies, 5% BSA was used for blocking. PVDF membrane were from Millipore, HRP-conjugated antibodies were from GE LifeSciences and chemiluminescence substrate was from Pierce.

DNA sequencing and sequence alignment

DNA sequencings were performed exactly according to the manufacturer’s protocol (BigDye Terminator Sequencing kit, Applied Biosystems).

Immunofluorescence

HA-, FLAG- or un-tagged NFIX expressing wild-type or HA-tagged mutant plasmids were transfected into U251 or 18KJ40 cells and immunofluorescence performed with HA (Chemiocon), FLAG (Sigma), CGGBP1 (mouse anti-human from ABCAM), CUGBP1 (mouse anti-human from ABCAM), TIA1 (rabbit anti-human from ABCAM), HMGN1 (rabbit anti-human from ABCAM) or NFIX antibodies. Cells were grown on coverslips, fixed using 4% formaldehyde for 10 minutes, permeabilized using PBS-0.1% Triton-X 100, blocked using 5% BSA and incubated with primary antibody overnight at 4ºC. Washings were performed in PBS-0.05% Triton-X 100 and secondary antibody (RRX-anti-rabbit) incubations were performed for 1 h at room temperature. After washings, coverslips were mounted on glass slides using DAPI containing mounting medium and analyzed under confocal microscope. Secondary antibody without primary antibody was used as negative control.

DNA sequence analysis

The ENSEMBL identifiers of the deregulated and control genes were used to retrieve -1 to -3000 bp long upstream sequences using the "retrieve-seq" program in RSA-tools (1,2) against the Homo Sapiens EnsEMBL database v. 47 (3).

The sequences obtained were then screened for the NFIX putative patterns (according to the ambiguous nucleotide codes of the IUPAC-IUB commission) using the "DNA-pattern" program in RSA-tools, default values were used and output with statistics selected.

PRIMERS AND REACTION CONDITIONS

All primers sequences are mentioned in Table S3.

**Supplementary references**

(1) van Helden J. Regulatory sequence analysis tools. Nucleic Acids Res. 2003 Jul 1;31(13):3593-6.

(2) Thomas-Chollier M et al. RSAT: regulatory sequence analysis tools. Nucleic Acids Res. 2008 Jul 1;36(Web Server issue):W119-27. Epub 2008 May 21.

(3) Flicek P et al. Ensembl 2008. Nucleic Acids Res. 2008 Jan;36(Database issue):D707-14. Epub 2007 Nov 13.
