# Supplementary figures and images for: A DNA Sequence Directed Mutual Transcription Regulation of HSF1 and NFIX Involves Novel Heat Sensitive Protein Interactions
Source: PLoS One. 2009 Apr 1;4(4):e5050. doi: 10.1371/journal.pone.0005050 (PMC2660424; doi:10.1371/journal.pone.0005050)

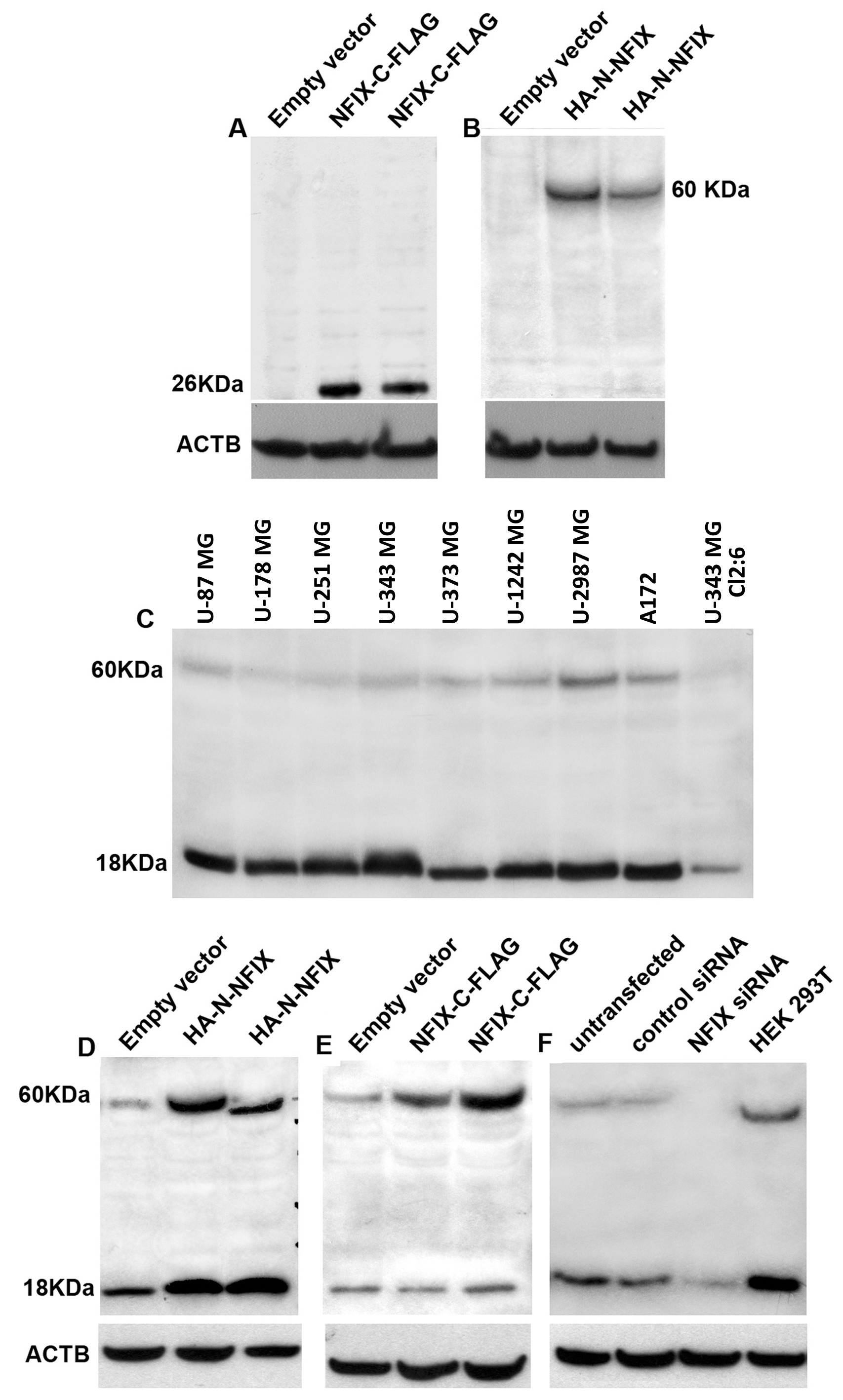

Supplement: Figure S1 — Different NFIX peptides: (A) C-terminal FLAG tag recognizes an 18 KDa peptide coded by NFIX-C-FLAG, which together with 8 KDa tag is recognized as a 26 KDa band. (B) N-terminal HA tag recognizes a 60 KDa peptide coded by HA-N-NFIX. (C) Rabbit anti-human NFIX antibody directed against C terminus of NFIX peptide recognizes both the peptides in nine different human glioma cell lines. U-2987 MG expresses highest and U-343 MG expresses lowest amounts of the 60 KDa peptide. Comparable protein loading in different lanes was confirmed by Coomassie blue staining (not shown). (D) HA-N-NFIX codes for both peptides as its transfections increase expression of both peptides as recognized by NFIX antibody. (E) NFIX antibody recognizes the 60 KDa peptide but not the 26 KDa peptide coded by NFIX-C-FLAG construct, perhaps due to epitope masking by FLAG tag. (F) NFIX-siRNA downregulates both peptides in U-2987 MG cells, confirming the identity of the two peptides as products of NFIX mRNA. The last lane shows NFIX expression in HEK 293T cells, used in transient transfection experiments. Two lanes for each construct shown in (A), (B), (D) and (E) represent the expression from two independent clones for each construct. (1.33 MB TIF) [file pone.0005050.s002.tif]

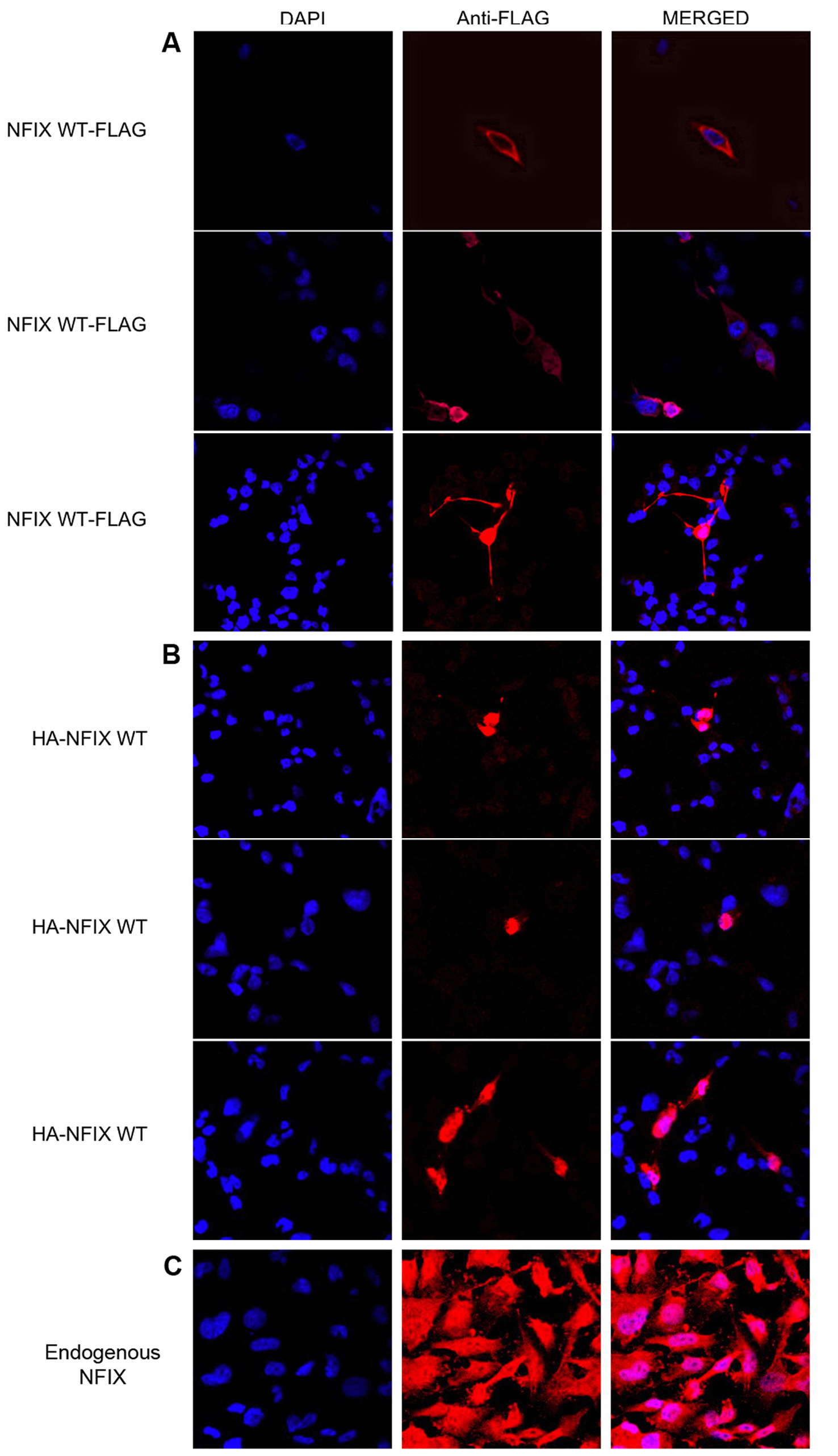

Supplement: Figure S2 — Expression of NFIX peptides in U-251 MG cells. (A) C-terminal FLAG tagged peptide is primarily present out of nucleus (top two panels). Rarely, it was seen strongly in nucleus also (bottom panel). (B) N-terminal HA-tagged peptide is nuclear in most cells (top two panels) and infrequently present in cytoplasm too (bottom panel). (C) NFIX antibody recognized endogenously expressed NFIX as present all over the cells. Staining with no primary antibody and pre-immune serum in case of NFIX antibody were used as negative controls. (2.38 MB TIF) [file pone.0005050.s003.tif]

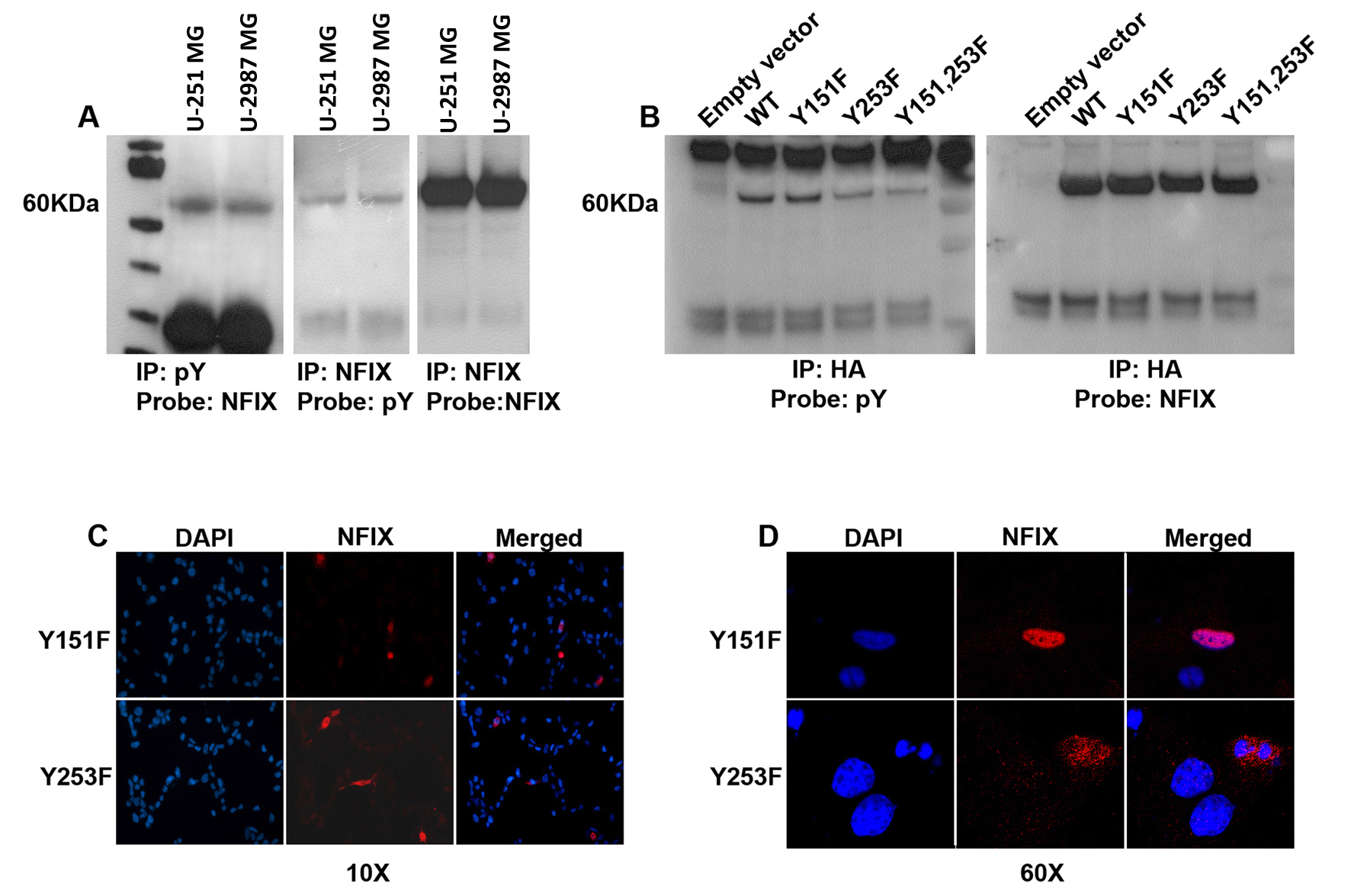

Supplement: Figure S3 — Tyrosine phosphorylation of NFIX. (A) Endogenously expressed NFIX is tyrosine phosphorylated in U-251 MG and U-2987 MG cells. (B) Transgenically expressed wild type (WT) and Y151F mutant constructs showed comparable levels of phosphorylation. But Y253F mutation was associated with reduction in phosphorylation levels with comparable levels of expression of NFIX peptide irrespective of the mutations. The HA antibody non-specifically recognized an approximately 90 KDa peptide. IP; immunoprecipitation, pY; phosphotyrosine antibody, Probe; the primary antibodu used in Western analysis following IP. (C) and (D); Y151F mutation does not affect intra-nuclear presence of HA-N-NFIX peptide (top panels of C and D); Y253F mutation leads to extra-nuclear presence of a fraction of HA-N-NFIX (lower panels of C and D). See text for details of the constructs. (1.21 MB TIF) [file pone.0005050.s004.tif]

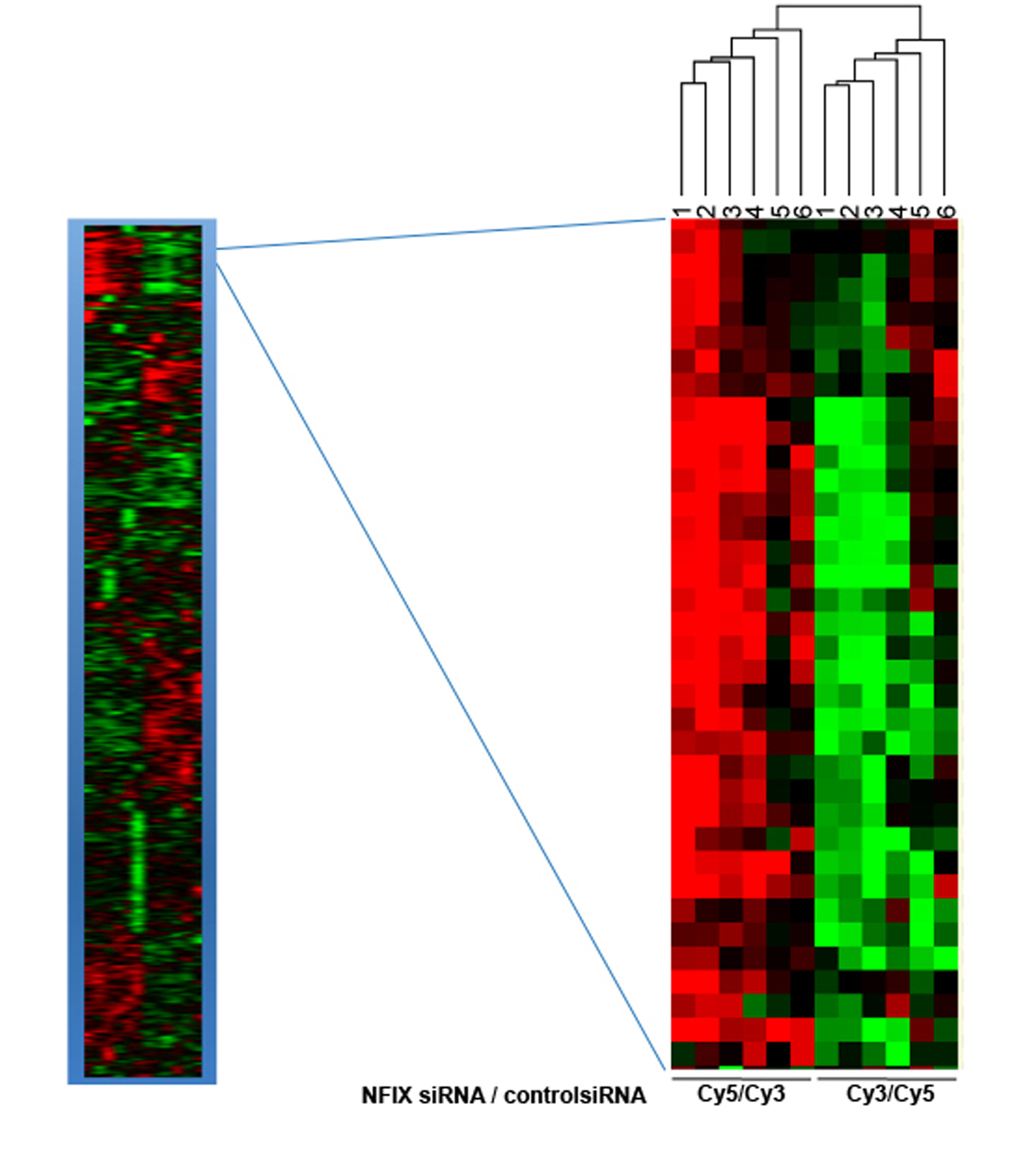

Supplement: Figure S4 — Heat-map plot of 12 microarray hybridizations show reproducibility of NFIX-siRNA-induced changes in global gene expression. The hybridizations represented by transfection number 6 are the most different from the remaining 5 for most of the reporters. (1.46 MB TIF) [file pone.0005050.s005.tif]

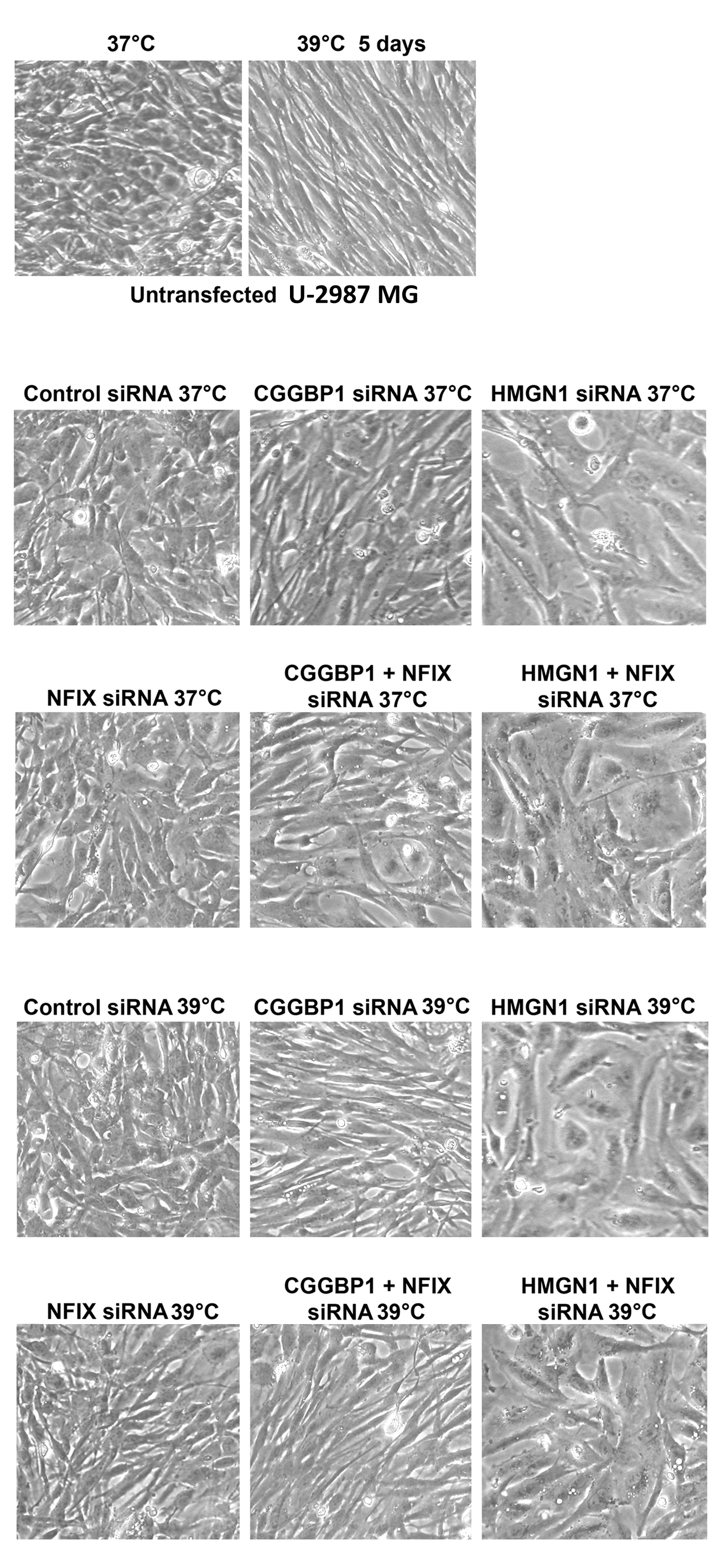

Supplement: Figure S6 — Phenotypes of U-2987 MG cells after chronic heat shock and/or siRNA indicated. CGGBP1-siRNA recapitulates chronic heat shock even at 37Â°C and HMGN1-siRNA leads to enlarged morbid cells. Combining either siRNA with NFIX-siRNA did not worsen the phenotypes. (2.09 MB TIF) [file pone.0005050.s007.tif]
