# Supplementary material for: A DNA Sequence Directed Mutual Transcription Regulation of HSF1 and NFIX Involves Novel Heat Sensitive Protein Interactions
Source: PLoS One. 2009 Apr 1;4(4):e5050. doi: 10.1371/journal.pone.0005050 (PMC2660424; doi:10.1371/journal.pone.0005050)
Supplement: Figure S5 — Sequence alignment of prey clones identified in the yeast-2-hybrid screen with RefSeq RNA sequences. (A) Two different clones of HMGN1 and (B) one clone of CGGBP1. Stars indicate exact match. (0.02 MB PDF) [file pone.0005050.s006.pdf]

Alignment between HMGN1 prey clone#1 and HMGN1 Human RefSeq sequence

```
Y2h_Clone_HMGN1_1      --------------------------------------ATATGGCCANGGAGGCCCCGGG
HMGN1_RefSeq           TTGTTTTTTAGTAGAATTGTTTCCTAAAGAAAACCACTCTTTGATCATGGCTCTCTCTG-
                                                               * **  ** **    * * * 

Y2h_Clone_HMGN1_1      GATCCGAATTCGCGGCCGCGTCG-ACCATCTTTGNGGTAGTCCNGTTTTCCTAATAACTT
HMGN1_RefSeq           ---CCAGAATTGTGTGCACTCTGTAACATCTTTGTGGTAGTCCTGTTTTCCTAATAACTT
                          **  * * * *  * *   * * ******** ******** ****************

Y2h_Clone_HMGN1_1      TGTTACNGNGCTGNGAAAGATTACAGATTTGAACATGTAGNGTACGTGCTGTTGAGTTGN
HMGN1_RefSeq           TGTTACTGTGCTGTGAAAGATTACAGATTTGAACATGTAGTGTACGTGCTGTTGAGTTGT
                       ****** * **** ************************** ****************** 

Y2h_Clone_HMGN1_1      GAACTGGNGGGCCGTATGTAACAGCTGACCAACGNGAANATACTGGTACTTGATAGCCTC
HMGN1_RefSeq           GAACTGGTGGGCCGTATGTAACAGCTGACCAACGTGAAGATACTGGTACTTGATAGCCTC
                       ******* ************************** *** *********************

Y2h_Clone_HMGN1_1      TTAAGGAAAATTTGCTTCCAAATTTTAAGCTGGAAAGTCACTGGAATAACTTTAAAAAAN
HMGN1_RefSeq           TTAAGGAAAATTTGCTTCCAAATTTTAAGCTGGAAAGTCACTGGAATAACTTTAAAAAAG
                       *********************************************************** 

Y2h_Clone_HMGN1_1      AATTACAATACANGGNTTTTTANAATTTCGTTACGTATGTTAAGATTTGNGTACAAATTG
HMGN1_RefSeq           AATTACAATACATGGCTTTTTAGAATTTCGTTACGTATGTTAAGATTTGTGTACAAATTG
                       ************ ** ****** ************************** **********

Y2h_Clone_HMGN1_1      AAATGTCTGTACNGATCCTCAACCAATAAAATCTCAGTTTATGAAAATAAAAAAAAAAAA
HMGN1_RefSeq           AAATGTCTGTACTGATCCTCAACCAATAAAATCTCAGTT-ATGAAAATAAAAAAAAAA--
                       ************ ************************** ******************  
```

Alignment between HMGN1 prey clone#2 and HMGN1 Human RefSeq sequence

```
Y2h_Clone_HMGN1_2      -TGTTCCANATTACGCTAGCTN-----GGGNGGTCATATGGCCNNNNAGGCCCCGGGGAT
HMGN1_RefSeq           TTGTTTTTTAGTAGAATTGTTTCCTAAAGAAAACCACTCTTTGATCATGGCTCTCTCTGC
                        ****    * **   * * *       *     **            *** *       

Y2h_Clone_HMGN1_2      CCGAATTCGCGGCCGCGTCGACCATNTTTGNGGTAGTCCTGTTTTCCTAATAACTTTGTT
HMGN1_RefSeq           CAGAATTGTGTGCACTCTGTAACATCTTTGTGGTAGTCCTGTTTTCCTAATAACTTTGTT
                       * *****    **    *  * *** **** *****************************

Y2h_Clone_HMGN1_2      ACTGNGCTGNGAAANATTACANATTTGAACATGTAGNGTACGTGCTGTTGAGTTGNGAAC
HMGN1_RefSeq           ACTGTGCTGTGAAAGATTACAGATTTGAACATGTAGTGTACGTGCTGTTGAGTTGTGAAC
                       **** **** **** ****** ************** ****************** ****

Y2h_Clone_HMGN1_2      TGGNGGGCCGTATGTAACAGCTGACCAACGNGAAGATNCNGGTACTNGATAGCCTCTTAA
HMGN1_RefSeq           TGGTGGGCCGTATGTAACAGCTGACCAACGTGAAGATACTGGTACTTGATAGCCTCTTAA
                       *** ************************** ****** * ****** *************

Y2h_Clone_HMGN1_2      GGAAAATTTGCTTCCAAATTTTAAGCTGGAAAGTCNCNGGAATAACTTTAAAAAANAATT
HMGN1_RefSeq           GGAAAATTTGCTTCCAAATTTTAAGCTGGAAAGTCACTGGAATAACTTTAAAAAAGAATT
                       *********************************** * ***************** ****

Y2h_Clone_HMGN1_2      ACAATACATGGNTTTTTANAATTTCGTTACGTATGTTAANATTTGNGTACAAATTGAAAT
HMGN1_RefSeq           ACAATACATGGCTTTTTAGAATTTCGTTACGTATGTTAAGATTTGTGTACAAATTGAAAT
                       *********** ****** ******************** ***** **************

Y2h_Clone_HMGN1_2      GTCTGTACTGATCCTCAACCAATAAAATCTCAGTTTATG---------------
HMGN1_RefSeq           GTCTGTACTGATCCTCAACCAATAAAATCTCAGTT-ATGAAAATAAAAAAAAAA
                       *********************************** ***
```

Alignment between CGGBP1 prey clone and CGGBP1 Human RefSeq sequence

```
Y2h_Clone_CGGBP1      GTCATATGGCCATGGAGGCCCCGGGGATCCNAATTCG---CGGCCGCGTCNACTTGGTAT
CGGBP1_RefSeq         CTGTTAGGATTATATCCTCTCCTTAGAAATGTTTTCCATCCTGTTGTGGGGATTTGGTAT
                       *  ** *   **     * **   **      ***    * *  * *   * *******

Y2h_Clone_CGGBP1      TAATGTTTCTTCATTGATTATGGAACTTTTGTTCCCTGAAGCTAGTTAACTGTGTCTAAA
CGGBP1_RefSeq         TAATGTTTCTTCATTGATTATGGAACTTTTGTTCCCTGAAGCTAGTTAACTGTGTCTAAA
                      ************************************************************

Y2h_Clone_CGGBP1      GTTAAATAAATACAGCANAANGATTGTTTTAGCCTGCTCGAACTGATACAAATCCCTGAA
CGGBP1_RefSeq         GTTAAATAAATACAGCAGAATGATTGTTTTAGCCTGCTCGAACTGATACAAATCCCTGAA
                      ***************** ** ***************************************

Y2h_Clone_CGGBP1      ACCATGATTGGCATATGTTANATAACAAATGAGGATGTCTAANAGGCATATGCTGCTTTG
CGGBP1_RefSeq         ACCATGATTGGCATATGTTAGATAACAAATGAGGATGTCTAAGAGGCATATGCTGCTTTG
                      ******************** ********************* *****************

Y2h_Clone_CGGBP1      GAGGNGTAGNGAACGTGNGTACAGAAGTTTTCAATCTTAACTATATAGNGTTAGNGNGAT
CGGBP1_RefSeq         GAGGTGTAGTGAACGTGTGTACAGAAGTTTTCAATCTTAACTATATAGTGTTAGTGTGAT
                      **** **** ******* ****************************** ***** * ***

Y2h_Clone_CGGBP1      GCTATACTATTGGAAAAATAGCAGNTTTTTTCTNTTTTATAAGTTGTATGCATAAACATA
CGGBP1_RefSeq         GCTATACTATTGGAAAAATAGCAGCTTTTTTCTATTTTATAAGTTGTATGCATAAACATA
                      ************************ ******** **************************

Y2h_Clone_CGGBP1      ANATTTGTAANGTTTCATTTATAAACTGCCTTCTTCAACACATGTTAATAGNGTTTTCTC
CGGBP1_RefSeq         AGATTTGTAATGTTTCATTTATAAACTGCCTTCTTCAACACATGTTAATAGTGTTTTCTC
                      * ******** **************************************** ********

Y2h_Clone_CGGBP1      AAAGTATTGATAGTATGTCTTCCANAATTTCACAATATGCTTACAGNAAATNTTTCCTAG
CGGBP1_RefSeq         AAAGTATTGATAGTATGTCTTCCAGAATTTCACAATATGCTTACAGTAAATATTTCCTAG
                      ************************ ********************* **** ********

Y2h_Clone_CGGBP1      NTNGTTNAAANGTT----------------------------------------------
CGGBP1_RefSeq         CTTGTTGAAATGTTCAATTCTTTGTTGGCTTTCTTCTTGATTCTGTGGGGGTGTATAACA
                       * *** *** ***
```
